# Age at menarche and adverse pregnancy and perinatal outcomes: triangulating evidence from multivariable and Mendelian randomization analyses

**DOI:** 10.1093/ije/dyag094

**Published:** 2026-06-24

**Authors:** Elisabeth Aiton, Maria Carolina Borges, Ana Gonçalves Soares, Gemma L Clayton, Tom A Bond, Qian Yang, Maria C Magnus, Deborah A Lawlor, Abigail Fraser, Amy E Taylor

**Affiliations:** Population Health Sciences, Bristol Medical School, Bristol, BS1 5DS, United Kingdom; MRC Integrative Epidemiology Unit, University of Bristol, Bristol, BS1 5DS, United Kingdom; Population Health Sciences, Bristol Medical School, Bristol, BS1 5DS, United Kingdom; MRC Integrative Epidemiology Unit, University of Bristol, Bristol, BS1 5DS, United Kingdom; Population Health Sciences, Bristol Medical School, Bristol, BS1 5DS, United Kingdom; MRC Integrative Epidemiology Unit, University of Bristol, Bristol, BS1 5DS, United Kingdom; Population Health Sciences, Bristol Medical School, Bristol, BS1 5DS, United Kingdom; MRC Integrative Epidemiology Unit, University of Bristol, Bristol, BS1 5DS, United Kingdom; Population Health Sciences, Bristol Medical School, Bristol, BS1 5DS, United Kingdom; MRC Integrative Epidemiology Unit, University of Bristol, Bristol, BS1 5DS, United Kingdom; Department of Endocrine and Metabolic Diseases, Shanghai Institute of Endocrine and Metabolic Diseases, Ruijin Hospital, Shanghai Jiao Tong University School of Medicine, Shanghai 200025, China; Shanghai National Clinical Research Center for Metabolic Diseases, Key Laboratory for Endocrine and Metabolic Diseases of the National Health Commission of the PR China, Shanghai Key Laboratory for Endocrine Tumor, Lifecycle Health Management Center, Ruijin Hospital Shanghai Jiao Tong University School of Medicine, Shanghai 200025, China; Centre for Fertility and Health, Norwegian Institute of Public Health, Oslo 0456, Norway; Population Health Sciences, Bristol Medical School, Bristol, BS1 5DS, United Kingdom; MRC Integrative Epidemiology Unit, University of Bristol, Bristol, BS1 5DS, United Kingdom; Population Health Sciences, Bristol Medical School, Bristol, BS1 5DS, United Kingdom; MRC Integrative Epidemiology Unit, University of Bristol, Bristol, BS1 5DS, United Kingdom; Population Health Sciences, Bristol Medical School, Bristol, BS1 5DS, United Kingdom; Department of Targeted Intervention, Division of Surgery and Interventional Science, University College London, London, WC1E 6BT, United Kingdom

**Keywords:** menarche, adiposity, body mass index, pregnancy, Mendelian randomization, multivariable Mendelian randomization, triangulation, ALSPAC

## Abstract

**Background:**

Observational studies have suggested that a younger age at menarche is associated with increased risks of adverse pregnancy and perinatal outcomes. However, it is unclear whether these relationships are causal.

**Methods:**

We estimated the associations between age at menarche and 13 pre-specified pregnancy outcomes by using two approaches. We estimated observational associations in the Avon Longitudinal Study of Parents and Children (*N* = 9441) using multivariable regression accounting for educational attainment, ethnicity, maternal age, parity, offspring sex, and adiposity. We conducted two-sample Mendelian randomization (MR) using data from the Mendelian Randomization in Pregnancy (MR-PREG) collaboration (77 683–707 797 pregnancies) and multivariable MR (MVMR) accounting for genetically-proxied adiposity.

**Results:**

Older age at menarche was associated with lower risks of hypertensive disorders of pregnancy, gestational hypertension, and preeclampsia, but accounting for adiposity attenuated these effects across approaches. For example, per 1-year older age at menarche, the odds ratio (OR) for hypertensive disorders of pregnancy was 0.88 (95% confidence interval (CI) : 0.84, 0.93) in inverse variance weighted MR and 0.95 (95% CI: 0.90, 1.01) in MVMR, while the observational association attenuated from OR = 0.91 (95% CI: 0.87, 0.94) to OR = 0.97 (95% CI: 0.93, 1.01). No clear evidence was found for the effects of age at menarche on small-for-gestational-age, low birthweight, post-term birth, or perinatal depression from either approach. For other outcomes evidence was limited by imprecision (very preterm birth, gestational diabetes) or inconsistent effects in sensitivity analyses (offspring birthweight, large-for-gestational-age, high birthweight, preterm birth).

**Conclusion:**

We find little robust evidence for causal effects of age at menarche on pregnancy outcomes. Effects of younger menarche on increased risks of hypertensive disorders of pregnancy may be driven by adiposity.

Key Messages

- We investigated whether reported associations between an earlier age at menarche and increased risks of some pregnancy complications are causal.
- We found that menarche timing was unlikely to affect risks of perinatal depression, post-term birth, or having a low-birthweight baby, while an adverse effect of earlier menarche on hypertensive disorders of pregnancy may be explained by elevated adiposity.
- Our findings argue against a substantial and direct causal effect of pubertal timing in the etiology of most pregnancy complications, but hint towards a potential role for childhood adiposity on the causal path for some.

## Background

Secular trends show a global decline in age at menarche (AAM) since the nineteenth century [1–3]. In northern Europe, the mean AAM declined from 16 years in the 1860s to 13 years in the early twenty-first century [3, 4]. Younger onset of female puberty is associated with multiple adverse health outcomes, including sex-steroid-sensitive cancers, cardiovascular disease, and depression [5–7], as is older onset [6, 7]. Studies indicate that women who experience menarche at a younger age have increased risks of adverse pregnancy and perinatal outcomes (APPOs), including hypertensive disorders of pregnancy (HDPs), gestational diabetes mellitus (GDM), and preterm birth [8–12].

It remains unclear whether AAM is a causal factor for APPOs and how adiposity may play a role. Estimated effects between AAM and APPOs could be confounded by childhood adiposity, which influences both puberty timing [13–15] and adult adiposity, the latter being a risk factor for several APPOs [16, 17]. However, younger puberty may also lead to increased adult adiposity and subsequent APPOs [13, 18]. Few studies of AAM–APPO relationships have investigated the role of adiposity; among observational studies accounting for adult body mass index (BMI), associations remained after adjustment in some [10–12] but not all [8, 9]. Understanding whether younger AAM causes APPOs could aid risk stratification and resolving APPO etiology to inform preventative treatments.

Our aim was to estimate the causal effect of AAM on APPOs by triangulating results from confounder-adjusted multivariable regression applied to a UK pregnancy cohort and two-sample Mendelian randomization (MR) [19]. If the results from both methods, subject to different sources of bias, show consistent direction and magnitude, then we can be more confident that the findings reflect a causal effect. In conventional observational analyses, residual confounding is a key source of bias. MR uses genetic variants as instruments for AAM to test unconfounded causal effects on APPOs [4]. As genetic variants are fixed at conception, MR analyses should mitigate confounding by factors such as socioeconomic position [20]. However, an important source of potential bias in MR analyses is horizontal pleiotropy. Here, this would be present if genetic instruments affected APPOs through pathways other than via AAM. To explore this, we conducted pleiotropy-robust MR sensitivity analyses [21–23] and multivariable MR (MVMR) accounting for any direct effects of childhood adiposity on APPOs.

## Methods

### Reporting and data availability

We followed STROBE [24] and STROBE-MR [25] reporting guidelines.

### Data sources

#### MR-PREG collaboration

We used data from the Mendelian Randomization in Pregnancy (MR-PREG) collaboration, which aims to understand the causes and consequences of APPOs [26]. This collaboration has harmonized APPOs and genetic data across five cohorts: the Avon Longitudinal Study of Parents and Children (ALSPAC) [27, 28], Born in Bradford [29, 30], FinnGen [31], the Norwegian Mother, Father and Child Cohort Study (MoBa) [32], and UK Biobank (UKB) [33]. ALSPAC was the only MR-PREG cohort used for observational analyses and is a British birth cohort that recruited pregnant women who were resident in Avon, UK with expected dates of delivery between 1991 and 1992 [27, 28, 34].

#### Summary statistics of genome-wide association studies

Published genome-wide association studies (GWASs) were used to select genetic instruments for AAM (see Supplementary Table S1 for GWAS details). Summary genetic association data for AAM were obtained from a GWAS of 632 955 participants [4]. For MVMR analyses, genetic instruments of childhood body size were identified from a GWAS of perceived body size at age 10 years [14] (*n* = 453 169 participants). Details are discussed in the Supplementary methods.

Associations between genetic instruments and outcomes were obtained from the MR-PREG collaboration [16, 26, 35] by combining data from all five cohort studies and publicly available GWASs [36–39]. GWASs were generated for all available outcomes in each study and then pooled in a fixed-effects inverse variance weighted meta-analysis (see collaboration profile [26] and the Supplementary methods for details).

### Exposure

Self-reported AAM in years was used as the exposure in both observational multivariable regression and MR analyses. In ALSPAC, we used the earliest available self-report across three assessments.

### Outcome measures

All APPO data were harmonized by using the MR-PREG collaboration definitions [26]. We considered the following APPOs: HDP, gestational hypertension (GH), preeclampsia, preterm birth, very preterm birth, small-for-gestational-age (SGA), low birthweight, GDM, post-term birth, large-for-gestational-age (LGA), high birthweight, perinatal depression, and continuous offspring birthweight (see Supplementary Table S2 for definitions and Supplementary Table S3A and B for total sample sizes). These outcomes were selected before analysis according to previous evidence [8–12, 40].

### Covariates

In ALSPAC, we selected covariates a priori that were potential confounders (categorical highest educational attainment, ethnicity coded as White/non-White) or that explained substantial variation in APPO risk (age at delivery, parity, and offspring sex). For full covariate definitions, see Supplementary Table S4.

Childhood adiposity was unavailable in ALSPAC, so we use self-reported pre-pregnancy BMI as the earliest available BMI measure. We expect this to be an adequate proxy of childhood adiposity; the robustness of this proxy was assessed in the ALSPAC offspring generation, in which BMI at age 9 years was strongly positively correlated with BMI at age 24 years (*r* = 0.62). As AAM has a small inverse effect on later-life adiposity [13], pre-pregnancy BMI may also mediate the effects of AAM on APPOs. We therefore present multivariable regression models both with and without adjustment for pre-pregnancy BMI and acknowledge that, where adjustment for pre-pregnancy BMI attenuates the associations, this might reflect over-adjustment rather than the confounder-adjusted result.

Although we account for different measures of adiposity due to practical constraints—childhood body size (MR) and pre-pregnancy BMI (multivariable regression)—we will refer to both as adiposity, for simplicity.

### Statistical analysis

#### Multivariable regression

We estimated the observational associations of AAM with APPOs by using logistic or linear regression. We present three models: an unadjusted model, a second model adjusting for confounders and covariates to ensure precision, and a third model accounting for all covariates and additionally adjusted for adiposity. See the Supplementary methods for details on sample selection, tests of linearity, and models exploring potential collider bias.

#### MR

Two-sample MR analysis was conducted to estimate the effect of the genetically-proxied AAM on APPOs (odds ratio or SD) using the TwoSampleMR R package (0.6.8) [41]. We selected independent single-nucleotide polymorphisms (SNPs; *R*^2^ < 0.001, 1000 Genomes European reference panel) associated with AAM at genome-wide significance (*P *< 5 × 10^−8^) as genetic instruments (see Supplementary Figure 1 for details). The primary analysis used the random-effects inverse variance weighted (IVW) method, selecting proxies for exposure SNPs in high linkage disequilibrium where necessary (*R*^2^ > 0.8). We identified 471 independent SNPs to instrument AAM.

MR analyses make three core assumptions (see Supplementary methods). We explored violations of these in several additional and sensitivity analyses. Instrument strength was evaluated by using mean F-statistics and total *R*^2^ [42]. As the most likely violation of the independence assumption is via population stratification, we used GWAS that were conducted in majority European-ancestry populations and accounted for population substructure by using linear mixed models and/or adjusting for principal components of ancestry [4, 14, 26]. We also tested for outlying studies within our APPOs GWAS in leave-one-study-out analyses.

We explored between-SNP heterogeneity by using Cochrane’s Q statistic. To assess the exclusion restriction assumption, we compared the MR IVW estimates to several univariable MR methods that are more robust to invalid instruments (i.e. “pleiotropy-robust”): MR-Egger [21], weighted median [22], and weighted mode [23] (details in the Supplementary methods).

Given that many genetic variants influencing AAM are also associated with childhood adiposity [4], the effects of AAM instruments could plausibly act via childhood adiposity to affect APPOs. We explored this potential violation of the exclusion restriction assumption by using MVMR to estimate the effects of AAM after accounting for childhood adiposity. We implemented analyses with the MVMR (0.4.2) R package [43]. MVMR was conducted by using 460 independent SNPs (see Supplementary Figure 2 for instrument selection details). Details of MVMR-specific measures of instrument strength and heterogeneity are provided in the Supplementary methods.

#### Sensitivity analyses

We performed (i) leave-one-study-out analyses to explore sample overlap and influential studies, (ii) analyses conditioned on fetal genotype to examine fetal genetic effects, and (iii) analyses using genetic variants mapped to genes with known biological roles in puberty onset. These analyses are described in detail in the Supplementary methods.

## Results

### Main analysis

The ALSPAC sample comprised 9441 singleton pregnancies to participants with data on self-reported AAM, at least one APPO, and all covariates. See Supplementary Figure 3 for a flow diagram and Supplementary Table S5 for sample sizes by APPO. The mean AAM was 12.9 years (1.5 SD) and the mean age at delivery was 28.5 years (4.80 SD). Women’s characteristics were generally similar across the early, intermediate, and late AAM categories (defined as below, within, and above 1 SD from the mean; Supplementary Table S6). However, women with an early AAM had a higher mean pre-pregnancy BMI (24.1, 4.9 SD) compared with women with intermediate (22.7, 3.6 SD) and late (22.2, 3.5 SD) AAM.

The 471 AAM SNPs explained 6% of the variance in AAM and had a mean F-statistic of 81 (range 30–1180). For the MVMR analysis, conditional F-statistics were 58 for AAM and 25 for adiposity.

**Figure 1:**
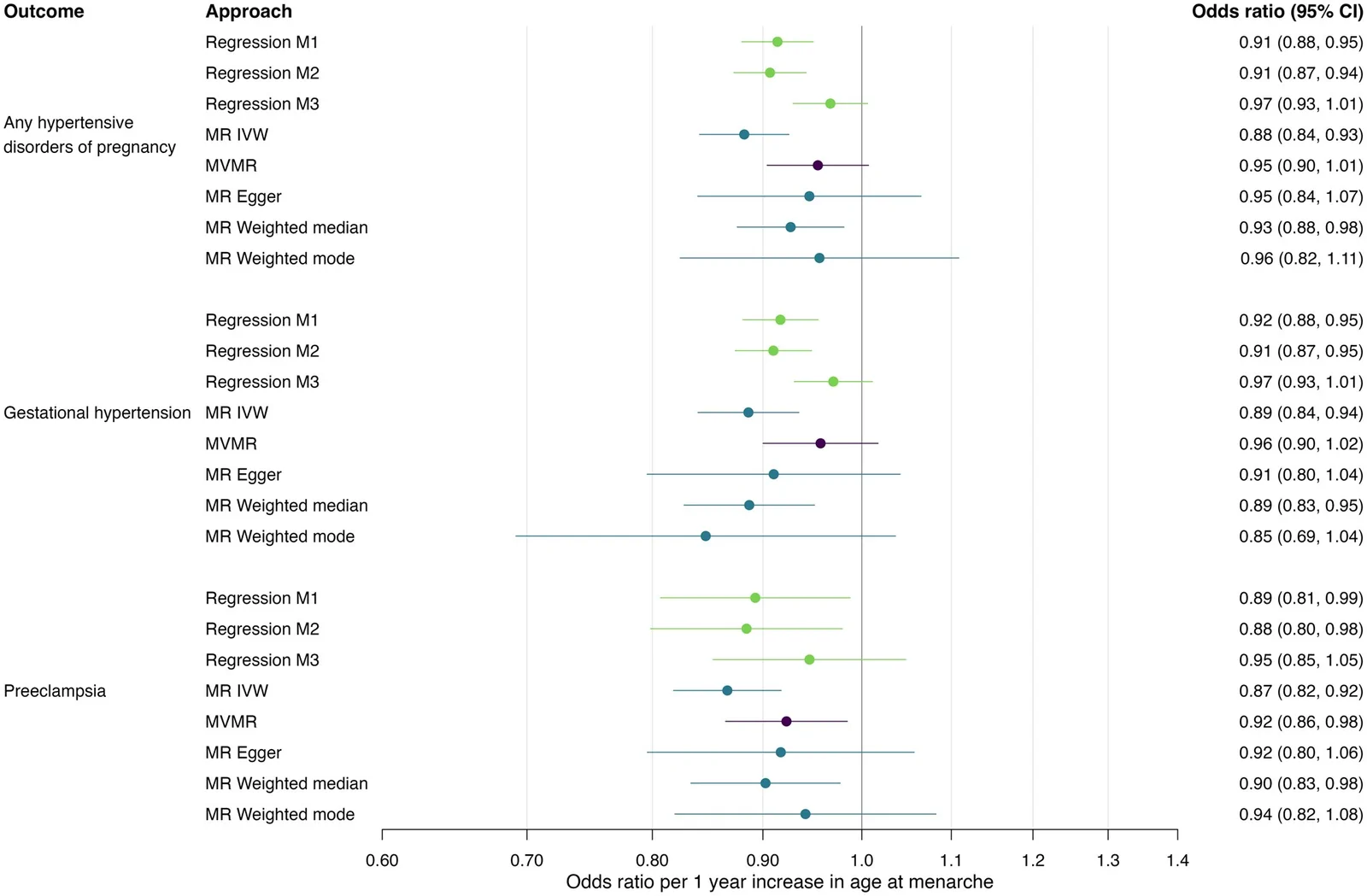
Observational multivariable regression and MR estimates for the effect of a 1-year increase in AAM on HDP. Illustrating (i) multivariable regression model 1 (M1): unadjusted estimate; (ii) multivariable regression model 2 (M2): estimate adjusted for highest educational attainment, ethnicity, age at delivery, parity, and offspring sex; (iii) multivariable regression estimate model 3 (M3): model 2 estimate with additional adjustment for adiposity; (iv) IVW MR estimate; (v) MVMR estimate accounting for adiposity; (vi) MR-Egger estimate; (vii) weighted median MR estimate; (viii) weighted mode MR estimate. Observational multivariable regression estimates are shown in green, univariable MR estimates are shown in blue, and MVMR estimates are shown in purple. MR = Mendelian randomization, AAM = age at menarche, HDP = hypertensive disorders of pregnancy, IVW = inverse variance weighted, MVMR = multivariable MR.

**Figure 2:**
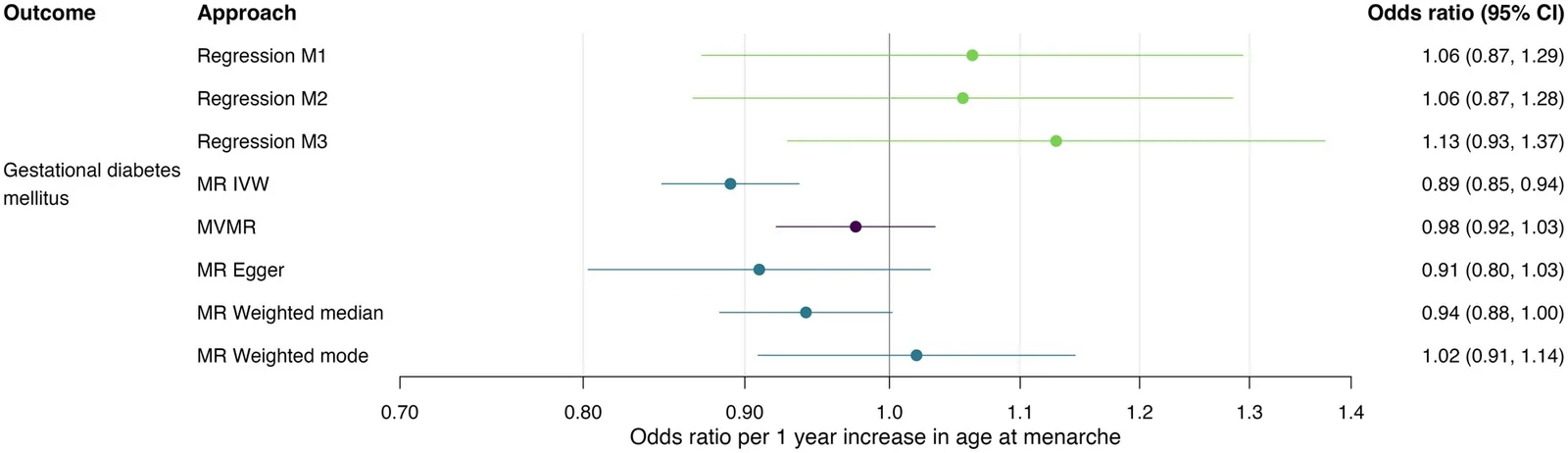
Observational multivariable regression and MR estimates for the effect of a 1-year increase in AAM on GDM. Illustrating (i) multivariable regression model 1 (M1): unadjusted estimate; (ii) multivariable regression model 2 (M2): estimate adjusted for highest educational attainment, ethnicity, age at delivery, parity, and offspring sex; (iii) multivariable regression estimate model 3 (M3): model 2 estimate with additional adjustment for adiposity; (iv) IVW MR estimate; (v) MVMR estimate accounting for adiposity; (vi) MR-Egger estimate; (vii) weighted median MR estimate; (viii) weighted mode MR estimate. Observational multivariable regression estimates are shown in green, univariable MR estimates are shown in blue, and MVMR estimates are shown in purple. MR = Mendelian randomization, AAM = age at menarche, GDM = gestational diabetes mellitus, IVW = inverse variance weighted, MVMR = multivariable MR.

**Figure 3:**
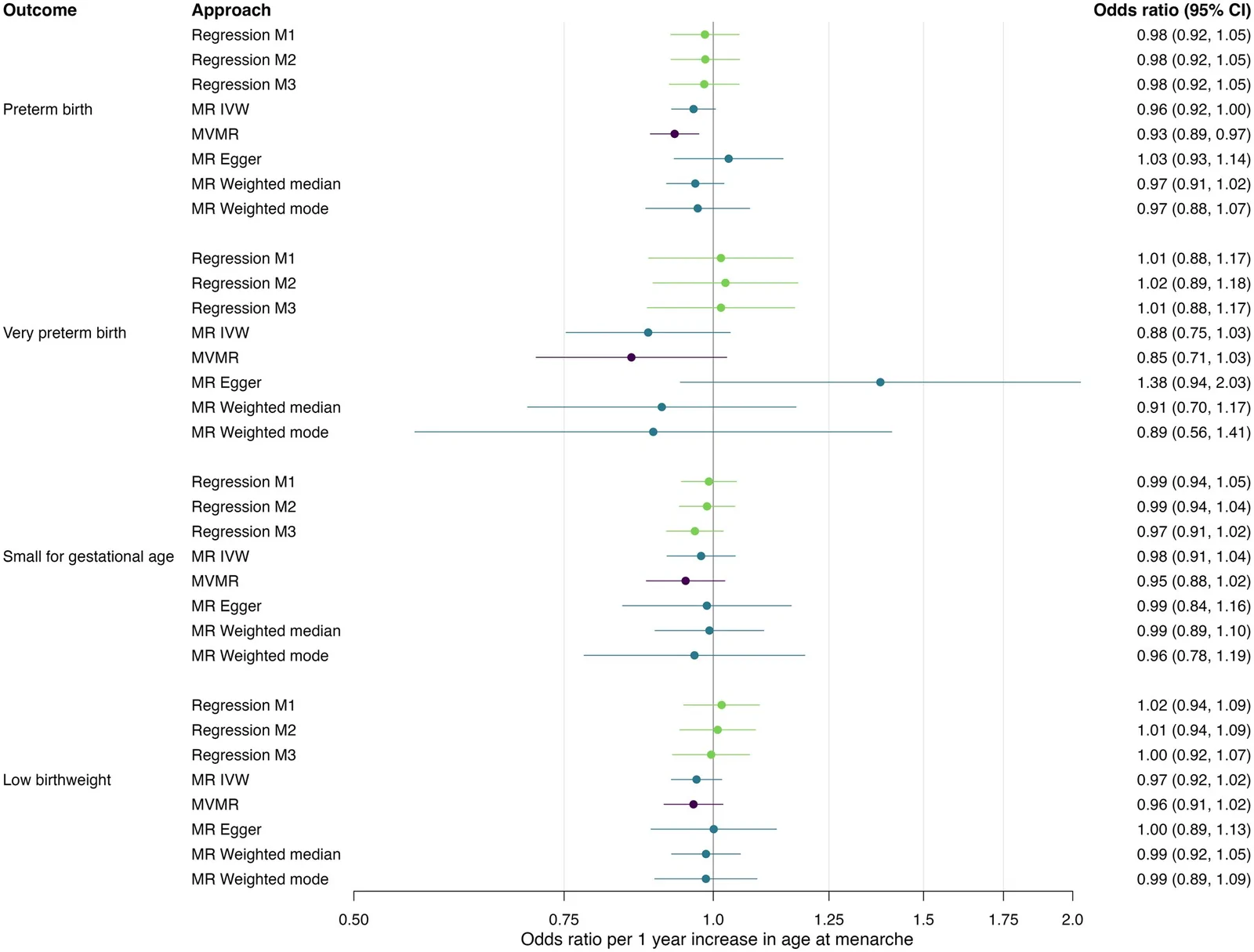
Observational multivariable regression and MR estimates for the effect of a 1-year increase in AAM on binary outcomes relating to preterm birth and small fetal size. Illustrating (i) multivariable regression model 1 (M1): unadjusted estimate; (ii) multivariable regression model 2 (M2): estimate adjusted for highest educational attainment, ethnicity, age at delivery, parity, and offspring sex; (iii) multivariable regression estimate model 3 (M3): model 2 estimate with additional adjustment for adiposity; (iv) IVW MR estimate; (v) MVMR estimate accounting for adiposity; (vi) MR-Egger estimate; (vii) weighted median MR estimate; (viii) weighted mode MR estimate. Observational multivariable regression estimates are shown in green, univariable MR estimates are shown in blue, and MVMR estimates are shown in purple. MR = Mendelian randomization, AAM = age at menarche, IVW = inverse variance weighted, MVMR = multivariable MR.

**Figure 4:**
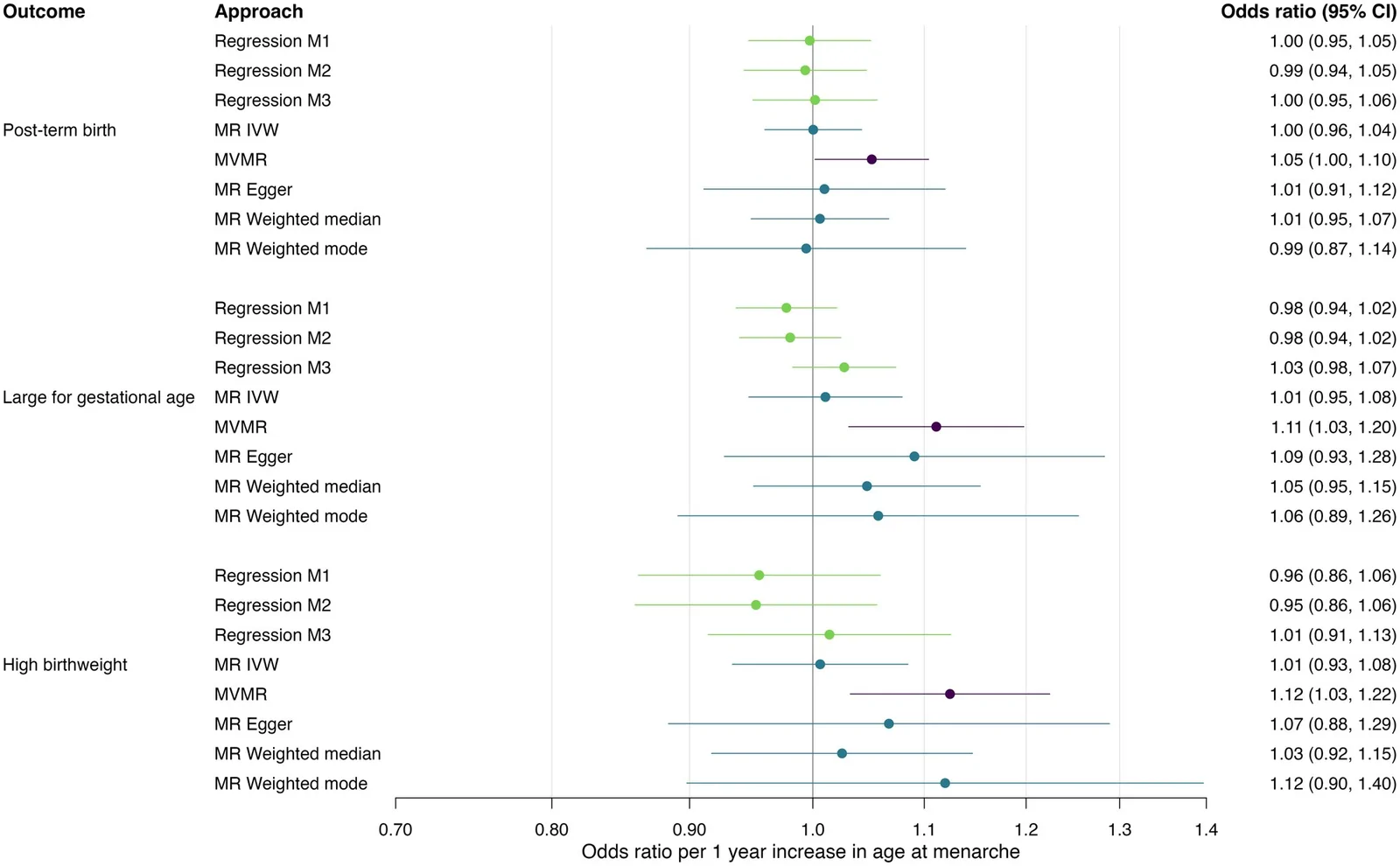
Observational multivariable regression and MR estimates for the effect of a 1-year increase in AAM on binary outcomes relating to post-term birth and large fetal size. Illustrating (i) multivariable regression model 1 (M1): unadjusted estimate; (ii) multivariable regression model 2 (M2): estimate adjusted for highest educational attainment, ethnicity, age at delivery, parity, and offspring sex; (iii) multivariable regression estimate model 3 (M3): model 2 estimate with additional adjustment for adiposity; (iv) IVW MR estimate; (v) MVMR estimate accounting for adiposity; (vi) MR-Egger estimate; (vii) weighted median MR estimate; (viii) weighted mode MR estimate. Observational multivariable regression estimates are shown in green, univariable MR estimates are shown in blue, and MVMR estimates are shown in purple. MR = Mendelian randomization, AAM = age at menarche, IVW = inverse variance weighted, MVMR = multivariable MR.

Figures 1–4 present the results of all multivariable regression models and MR analyses, using IVW and pleiotropy-robust estimators, and MVMR. The MR-Egger intercepts and Cochran’s Q-statistics are presented in full in Supplementary Tables S7 and S8, respectively.

#### HDP

In observational multivariable regression without adjustment for adiposity, older AAM was associated with a lower risk of any HDP (odds ratio (OR)  = 0.91 per 1-year older AAM, 95% confidence interval (CI): 0.87, 0.94; Fig. 1), GH (OR = 0.91, 95% CI: 0.87, 0.95), and preeclampsia (OR = 0.88, 95% CI: 0.80, 0.98). All estimates attenuated once adiposity was accounted for (HDP OR = 0.97, 95% CI: 0.93, 1.01; GH OR = 0.97, 95% CI: 0.93, 1.01; preeclampsia OR = 0.95, 95% CI: 0.85, 1.05).

In MR IVW analyses, a 1-year older genetically-proxied AAM was associated with a lower risk of any HDP (OR = 0.88, 95% CI: 0.84, 0.93), GH (OR = 0.89, 95% CI: 0.84, 0.94), and preeclampsia (OR = 0.87, 95% CI: 0.82, 0.92). Between-SNP heterogeneity was high for all these outcomes (Cochran’s Q-statistics *P *< 1 × 10^−21^) but the MR-Egger intercepts did not indicate directional horizontal pleiotropy. The estimates from MR-Egger, weighted median, and weighted mode analyses were broadly consistent but attenuated somewhat for HDP and preeclampsia.

In MVMR, the lower risks of HDP, GH, and preeclampsia with older genetically-proxied AAM observed in the MR IVW analysis were all attenuated towards the null (HDP OR = 0.95, 95% CI: 0.90, 1.01; GH OR = 0.96, 95% CI: 0.90, 1.02; preeclampsia OR = 0.92, 95% CI: 0.86, 0.98).

#### GDM

There was no evidence that AAM was associated with GDM in observational analyses, though estimates were very imprecise (Fig. 2). In MR IVW, women with an older genetically-proxied AAM had a lower risk of GDM (OR = 0.89, 95% CI: 0.85, 0.94) but there was weaker evidence for any effect from pleiotropy-robust methods (e.g. weighted mode OR = 1.02, 95% CI: 0.91, 1.14) and MVMR (OR = 0.98, 95% CI: 0.92, 1.03). Between-SNP heterogeneity was high for GDM (Cochran’s Q-statistics *P *< 1 × 10^−22^) but MR-Egger intercepts showed no evidence of directional horizontal pleiotropy.

#### Fetal size and gestational age at delivery

There was no strong evidence in observational analyses or MR IVW for an association between AAM and risks of earlier gestational age at delivery and smaller fetal size (Fig. 3; preterm birth, very preterm birth, SGA, or low birthweight) or between AAM and risks of later gestational age at delivery and larger fetal size (Fig. 4; post-term birth, LGA, or high birthweight). The estimated effects for very preterm birth were imprecise.

Between-SNP heterogeneity was moderately high for all outcomes (Cochran’s Q-statistics *P *< 1 × 10^−4^) except for SGA and very preterm birth. The MR-Egger intercepts indicated moderate directional horizontal pleiotropy for very preterm birth only (intercept = −0.015, *P *= .013) and the MR-Egger slope estimate was in the opposite direction to those of all other analyses.

Results from MVMR, MR-Egger, weighted median, and weighted mode estimators gave estimates that were consistent with those from MR IVW analyses for most outcomes. However, the results from these pleiotropy-robust methods were compatible with an older genetically-proxied AAM being associated with increased risks of LGA (e.g. MVMR OR = 1.11, 95% CI: 1.03, 1.20) and high birthweight (e.g. MVMR OR = 1.12, 95% CI: 1.03, 1.22).

Initial (unadjusted) analyses showed no clear association between AAM and continuous birthweight. However, accounting for adiposity in both observational analyses and multivariable MVMR revealed positive associations between AAM and birthweight (Supplementary Figure 4).

#### Perinatal depression

There was no clear evidence in observational analyses, MR IVW, or pleiotropy-robust MR methods for associations between AAM and perinatal depression (Supplementary Figure 5).

### Sensitivity analyses

Observational multivariable regression models considering AAM in categories generated results in line with the main findings (Supplementary Figures S6 and S7). Results excluding variables that could induce possible collider bias were very similar to those of the main analyses (Supplementary Figures S8 and S9).

Sample overlap of the AAM-exposure GWAS with the meta-analysed APPO GWAS ranged from 0% to 32.8%, varying by outcome (Supplementary Table S9), while, for the UKB adiposity GWAS, it ranged from 0% to 24.0% (Supplementary Table S10). Leave-one-study-out analyses suggested that the findings from MR analyses were not driven by overlapping or specific studies for most outcomes (Supplementary Figures S10 and S11, and Supplementary Table S11). The only exception was evidence that MoBa may drive the effect of genetically-proxied AAM on high birthweight in MVMR (without MoBa: OR = 1.02, 95% CI: 0.92, 1.14).

Utilizing an outcome GWAS adjusted for fetal genotype resulted in similar MR IVW estimates (Supplementary Figure 12 and Supplementary Table S11) and generally similar MVMR estimates (Supplementary Figure 13), though precision was reduced. We found some evidence that the MVMR estimates were attenuated when accounting for fetal genotype compared with the main analysis for LGA (MVMR accounting for fetal genotype: OR = 1.07, 95% CI: 0.98, 1.17), high birthweight (OR = 1.05, 95% CI: 0.94, 1.18), and birthweight (beta = 0.006, 95% CI: –0.014, 0.026) (Supplementary Table S12).

After adjustment for fetal genotype, the between-SNP heterogeneity was reduced in the MVMR analyses for all outcomes (Supplementary Table S13). However, there was still evidence of heterogeneity for HDP, GH, preeclampsia, preterm birth, and continuous birthweight (*P *< .001).

We identified six SNPs mapped to genes with known biological roles in pubertal timing (mean F-statistic = 113, *R*^2^ = 0.11%; Supplementary Table S14). The estimated effects when using this genetic instrument were compatible with the main IVW findings, but were less precise (Supplementary Figures S14 and S15).

## Discussion

We triangulated results from observational multivariable regression and MR approaches to investigate whether AAM causally influences a range of APPOs. Although we found that older AAM was associated with a reduced risk of HDP, once adiposity was accounted for in both multivariable regression and MVMR analyses, the associations were attenuated, suggesting that confounding by childhood adiposity was at play. In both observational multivariable regression and MR, we found consistent evidence of null effects on SGA, low birthweight, post-term birth, and perinatal depression. For other outcomes, the evidence was limited by imprecision or inconsistency in sensitivity analyses examining adiposity and horizontal pleiotropy.

Our findings for HDP are consistent with those of other studies [7, 8, 44, 45]. For example, one study found that the risk of HDP in women with an early AAM (8–11 years compared with 13 years) attenuated from 1.11 (95% CI: 0.97, 1.27) to 1.05 (95% CI: 0.92, 1.20) after accounting for a range of adulthood adiposity and body-composition measurements [7]. On the other hand, another recent study found that adjustment for early-pregnancy BMI did not alter the modest effects of younger AAM on increased risks of GH or preeclampsia [without-adjustment OR = 1.05 per 1-year decrease in AAM (95% CI: 1.03, 1.07), with-adjustment OR = 1.05 (95% CI: 1.03, 1.08)] [12]. Though the attenuation varies between studies, the confidence intervals are largely compatible and the magnitude of any effect is consistently modest. If the effect of AAM on HDP is wholly confounded by adiposity, then we may have found only a partial attenuation for two reasons. For observational analyses, we used a proxy measure of childhood adiposity (although strongly correlated); previous cohort studies have also used adulthood-adiposity measures. In our MVMR analysis, the instrument for AAM was substantially stronger than that for adiposity, so there may be some residual effects of adiposity that we have not accounted for. Our MR IVW and MVMR results suggest that the inverse association between AAM and risk of GDM may be also explained by adiposity. This contrasts with a US cohort study which found that adjustment for childhood adiposity did not attenuate the association between younger AAM and greater GDM risk [46]. To our knowledge, no other studies of this relationship have accounted for childhood adiposity [47]. The differences in our findings may be due to chance, residual confounding by adiposity in the US study, or power limitations in our analyses. Our observational analysis of GDM was underpowered, as GDM is likely under-diagnosed due to the absence of universal screening in the UK. We found stronger evidence for some AAM–outcome relationships in the MVMR than in the MR IVW analyses, e.g. for LGA. This could reflect a positive direct effect of later AAM, which is masked by an inverse indirect effect mediated via adiposity in MR IVW—as has been shown previously for breast cancer risk [48].

The key strength of this study is the triangulation across observational multivariable regression and MR approaches. Sources of potential bias differ between these approaches, including residual confounding in multivariable regression and horizontal pleiotropy in MR, such that consistent findings across both approaches provides stronger confidence in findings. Moreover, we were able to conduct sensitivity analyses adjusting for fetal genetics in MR analyses and to provide evidence on a comprehensive range of APPOs [49].

An important limitation was that we used pre-pregnancy BMI as a proxy for childhood adiposity in our observational analysis. This may have resulted in over-adjustment, as adult BMI is a plausible mediator of any effect of AAM on APPOs; AAM has a small inverse effect on adult BMI [13] and adult BMI affects the risks of numerous APPOs [16, 17].

The total sample sizes were small for very preterm birth (both approaches) and GDM (observational), which likely limited our power to detect the effects on these outcomes. We also used self-reported measures of AAM in adulthood as exposures in both approaches, which may not have been recalled accurately [50]. However, categorizing AAM can improve validity [50] and we found that this gave similar results in sensitivity analyses.

In the observational analysis, both measurement error and residual confounding may have biased our analyses, which could mean we have underestimated the effect of adiposity. The MR analyses were subject to several limitations. Our MVMR analysis accounted for pleiotropy via adiposity only, selected a priori, so it is possible that other pleiotropic pathways exist; we addressed this through triangulation with hypothesis-free pleiotropy-robust methods. Sample overlap between exposure and outcome GWASs may have biased our MR estimates towards the (confounded) observational associations [51], though leave-one-study-out analyses excluding the overlapping cohorts were consistent. Self-reported adiposity may have been subject to misclassification to a lower body size due to social desirability bias. Reassuringly, previous simulations of MVMR using this childhood body-size GWAS [14] suggested that this misclassification would not bias estimates for a second correlated exposure (here, AAM). Our analysis was restricted to cohorts of predominantly European ancestry, which may limit the generalizability of our findings to women of other ancestries.

## Conclusion

This study provides useful evidence on the etiology of a wide range of APPOs, many of which are poorly understood. We find limited evidence for any effects of AAM on APPOs and that reported associations between a younger AAM and risks of HDP may be explained by adiposity.

## Ethics approval

The pre-specified analysis plan and all analytical code are available online (https://github.com/eaiton/menarche_pregnancy). All analyses were performed by using R software version 4.3.3. Cohort-specific ethics approval details are provided in the Supplementary material.

## Supplementary Material

dyag094_Supplementary_Data

## Data Availability

Publicly available GWAS data used in this study have been referenced throughout the manuscript, including the GWAS for AAM and childhood body size. Genetic instruments used in the MR analyses are provided online (https://github.com/eaiton/menarche_pregnancy). To protect participant confidentiality, supporting outcome data cannot be made openly available. Researchers can apply for access to individual study executive committees. The ALSPAC access policy describes the proposal process in detail, including any costs associated with conducting research at ALSPAC, and may be updated from time to time (https://www.bristol.ac.uk/media-library/sites/alspac/documents/researchers/data-access/ALSPAC_Access_Policy.pdf). The ALSPAC study website contains details of all the data that are available through a fully searchable data dictionary and variable search tool (http://www.bristol.ac.uk/alspac/researchers/our-data/). Data are available upon request from Born in Bradford (https://borninbradford.nhs.uk/our-data/how-to-access-data). Data from MoBa are available from the Norwegian Institute of Public Health after application to the MoBa Scientific Management Group (see its website at https://www.fhi.no/en/ch/studies/moba/for-forskere-artikler/research-and-data-access for details). Researchers can apply for access to the UK Biobank data via the Access Management System (AMS) (https://www.ukbiobank.ac.uk/use-our-data/apply-for-access). FinnGen genetic summary statistics are freely available online (https://www.finngen.fi/en/access_results).
