# Supplementary material for: Age at menarche and adverse pregnancy and perinatal outcomes: triangulating evidence from multivariable and Mendelian randomization analyses
Source: Int J Epidemiol. 2026 Jun 24;55(4):dyag094. doi: 10.1093/ije/dyag094 (PMC13291527; doi:10.1093/ije/dyag094)
Supplement: dyag094_Supplementary_Data [file dyag094_supplementary_data.zip › ije-2025-09-1838-File006.pdf]

<u>Supplementary Material: text</u>

Table of Contents

Supplementary methods 2

Data sources 2

Childhood body size GWAS 2

Pregnancy outcome GWAS meta-analysis 2

Multivariable regression 2

Sample selection 2

Tests of linearity 2

Collider bias 2

Mendelian randomization 3

Core assumptions 3

Pleiotropy-robust methods 3

Multivariable Mendelian randomization 3

Sensitivity analyses 3

Cohort acknowledgments 5

ALSPAC 5

BiB 5

MoBa 5

FinnGen 5

UK Biobank 5

Cohort funding 6

ALSPAC 6

MoBa 6

BiB 6

UK Biobank 6

Cohort ethics approvals 7

ALSPAC 7

BiB 7

MoBa 7

UK Biobank 7

References 8

Supplementary methods

Data sources

Childhood body size GWAS

Genetic instruments for childhood body size were identified from a GWAS of perceived body size at age 10 in UK Biobank[1]. For this variable, participants reported if they were 'thinner', 'plumper' or 'about average' at age 10. We used this GWAS[1] rather than any of measured BMI in childhood since the sample size is larger. This measure has been extensively validated against BMI; the largest external replication to date in the Trøndelag Health Study (HUNT) found that the sex-combined polygenic risk score explained 6.7% of variance in childhood BMI (n=66,963)[2]. We used sex-combined childhood body size instruments since these explain more variance than instruments derived in female-only samples[3] and no sex-dependent genetic effects on BMI have been identified[4] (**Figure S16**).

Pregnancy outcome GWAS meta-analysis

GWAS meta-analyses for each pregnancy were outcome were generating by combining data from all five cohort studies and publicly available GWAS of GDM[5], preeclampsia[6], preterm birth[7], gestational duration[7] and perinatal depression[8]. We excluded cohorts which overlapped with publicly available GWAS or where 50 or fewer cases were available. Meta-analyses were conducted using METAL (v. 2020-05-05) [9]. For details of quality control and cohort-specific pipelines please see collaboration profile[10].

Multivariable regression

Sample selection

We restricted our ALSPAC sample to singleton pregnancies to unique mothers by randomly selecting one pregnancy. Complete case analysis was used for each APPO, assuming missingness was independent of the outcome after accounting for covariates[11].

Tests of linearity

We detected no evidence of departure from linearity in exposure-outcome relationships in likelihood ratio tests comparing nested models of early, intermediate, and late categories of AAM (defined as below, within, and above one standard deviation from the mean in our ALSPAC sample; **Table S15**). We used restricted cubic splines to additionally visualise these relationships (**Figures S17-18)**. We selected knot points *a priori* at the boundaries between early-intermediate (11 years) and intermediate-late (14 years) AAM categories, and adjusted for highest educational attainment, ethnicity, age at delivery, parity, offspring sex, and pre-pregnancy BMI. These were largely compatible with linear relationships, with the exceptions of large-for-gestational-age and post-term birth, though these were both subject to substantial imprecision. We therefore present results for AAM as a continuous variable (years) as the main analysis.

Collider bias

In observational cohort studies, AAM is positively correlated with age at delivery and with parity [12]. There is also evidence from Mendelian randomization that an older AAM may be causally related to an older age at delivery, and though there is less evidence for a causal relationship between older AAM and increased parity this has not been widely investigated [12]. Since both age at delivery and parity are important determinants of the risk of pregnancy outcomes, if these variables are caused by AAM and affected by unmeasured confounders of these pregnancy outcomes, adjusting for them could induce collider bias in our multivariable regression analyses (adjusted models 'M2' and 'M3' in Figures 1-4). To explore the extent of potential collider bias, we estimated the effect of AAM on all pregnancy outcomes without adjustment for age at delivery or parity (**Figures S8-S9**). In one model we estimated effects after adjusting for highest educational attainment, ethnicity, and offspring sex only; in a second model we included the same variables and additionally adjusted for adiposity.

Mendelian randomization

Core assumptions

Our MR analyses may be biased if three key assumptions are violated: (i) the instrument is strongly associated with AAM (relevance assumption), (ii) there is no confounding of the genetic instrument-APPO association (independence assumption), and (iii) the genetic instrument affects APPOs only through AAM (exclusion restriction assumption). In most MR studies, violation of this third assumption is likely the key source of bias.

Pleiotropy-robust methods

MR-Egger provides an estimate corrected for the presence of potential directional horizontal pleiotropy by adding an intercept term to the IVW model; we also considered the magnitude and statistical support for the MR-Egger intercept to assess evidence of such pleiotropy[13]. The weighted median and weighted mode estimators provide unbiased estimates when valid genetic instruments comprise at least 50% of the instrument weights or when the most common amount of horizontal pleiotropy is zero, respectively.

Multivariable Mendelian randomization

For multivariable Mendelian randomization (MVMR) analyses, we estimated instrument strength via Sanderson–Windmeijer conditional F-statistics[14], and between-SNP heterogeneity using a modified form of Cochrane's Q statistic[15]. To calculate these MVMR-specific F- and Q- statistics, we used previously described estimators[14,15] and estimated phenotypic covariance between exposures in the ALSPAC offspring generation (BMI at age 9 and AAM R=-0.301, n=3,669).

After clumping the 471 SNPs instrumenting AAM and 231 SNPs instrumenting adiposity, we used 460 independent SNPs for MVMR analyses (provided online[16]).

Sensitivity analyses

Sample overlap between AAM and APPOs GWAS were quantified, as overlap can bias both MR [17] and MVMR estimates[1]. Since UKB and ALSPAC were overlapping cohorts between the AAM and APPO GWAS, we considered effects of leaving out both cohorts in the APPO GWAS alongside the study-level leave-one-out meta-analyses.

Fetal genetic confounding could bias our estimates if maternal AAM genetic instruments affect offspring outcomes through genetic inheritance, rather than through an intrauterine causal effect of maternal AAM (i.e. violating the exclusion restriction assumption). To assess this, MR and MVMR results were replicated using SNP-outcome estimates obtained from APPO GWAS conditioned on fetal genotype using a weighted linear model[18], implemented in the DONUTS R package[19].

To explore potential bias due to horizontal pleiotropy, we replicated univariable MR estimates using a smaller subset of genetic variants with strong biological plausibility. Variant-to-gene mapping of AAM-associated variants was previously conducted [20]. This strategy used several molecular traits to rank the variant-gene pairs most likely to be biologically related to AAM, such as variant-gene proximity, eQTL and pQTL colocalisation, and evidence for coding variants in linkage disequilibrium. We selected the subset of our genetic instruments which had been mapped to genes in pathways with strong biologically supported roles in puberty onset. These genes were from three pathways, selected *a priori*: 1) genes with roles in the hypothalamic–pituitary–gonadal axis which are disrupted in monogenic disorders of puberty (*CADM1*, *CHD4*, *CHD7*, *FEZF1*, *GNRH1*, *KISS1*, *SPRY4*, *TAC3*, *TACR3* and *TYRO3*), 2) genes with roles in gametogenesis and sex hormone secretion (*ACVR2A, CYP19A1, HSD17B7, INHBA, INHBB, MC3R* and *PCSK2*), and 2) genes which are disrupted in syndromes characterised by hypogonadism (*BRAF*, *SOS1*, *BBS4, NDN*, *SNRPN* and *UBE3A*)[20]. We then conducted MR IVW using only genetic

variants which had been mapped to these genes, where these were available. Details of genetic variants used in this analysis are provided in **Table S14**.

Cohort acknowledgments

ALSPAC
We are extremely grateful to all the families who took part in this study, the midwives for their help in recruiting them, and the whole ALSPAC team, which includes interviewers, computer and laboratory technicians, clerical workers, research scientists, volunteers, managers, receptionists and nurses.

BiB
BiB is only possible because of the enthusiasm and commitment of the Children and Parents in BiB. We are grateful to all the participants, teachers, school staff, health professionals and researchers who have made BiB happen.

MoBa
This research has been conducted using MoBa data using application number 2552. MoBa is supported by the Norwegian Ministry of Health and Care services and the Ministry of Education and Research. We are grateful to all the participating families in Norway who take part in this on-going cohort study. We thank the Norwegian Institute of Public Health (NIPH) for generating high-quality genomic data. This research is part of the HARVEST collaboration, supported by the Research Council of Norway (#229624). We also thank the NORMENT Centre for providing genotype data, funded by the Research Council of Norway (#223273), South East Norway Health Authority and KG Jebsen Stiftelsen. We further thank the Center for Diabetes Research, the University of Bergen for providing genotype data and performing QC and imputation of the data funded by the ERC AdG project SELECTionPREDISPOSED, Stiftelsen Kristian Gerhard Jebsen, Trond Mohn Foundation, the Research Council of Norway, the Novo Nordisk Foundation,
the University of Bergen, and the Western Norway health Authorities (Helse Vest).

FinnGen
The authors thank the FinnGen investigators for sharing their summary-level data.

UK Biobank
We would like to thank all the participants of UK Biobank for their vital contribution to the resource. This research has been conducted using the UK Biobank Resource under Application Number 23938.

Cohort funding

ALSPAC
The UK Medical Research Council and Wellcome (Grant ref: 217065/Z/19/Z) and the University of Bristol provide core support for ALSPAC.A comprehensive list of grants funding is available on the ALSPAC website (http://www.bristol.ac.uk/alspac/external/documents/grant-acknowledgements.pdf). ALSPAC GWAS data was generated by Sample Logistics and Genotyping Facilities at Wellcome Sanger Institute and LabCorp (Laboratory Corporation of America) using support from 23andMe. This research was funded in part by the Wellcome Trust (Grant ref: 228276/Z/23/Z)]. For the purpose of Open Access, the author has applied a CC BY public copyright licence to any Author Accepted Manuscript version arising from this submission.

MoBa
MoBa funding is under Acknowledgements as requested by MoBa publication guidelines.

BiB
BiB is supported by a Wellcome Longitudinal Population Study Grant (223601/Z/21/Z); a joint grant from the UK Medical Research Council (MRC) and UK Economic and Social Science Research Council (ESRC) (MR/N024391/1); the British Heart Foundation (CS/16/4/32482); a Wellcome Infrastructure Grant (WT101597MA); the National Institute for Health Research under its Applied Research Collaboration for Yorkshire and Humber (NIHR200166). The National Institute for Health Research Clinical Research Network provided research delivery support for this study. The views expressed in this publication are those of the authors and not necessarily those of the National Institute for Health Research or the Department of Health and Social Care.

UK Biobank
UK Biobank is funded primarily by the Wellcome Trust and the Medical Research Council (MRC). It is also funded by the Department of Health, British Heart Foundation, Cancer Research UK, Diabetes UK, National Institute for Health Research (NIHR), Scottish Government, Northwest Regional Development Agency, and Welsh Assembly Government.

Cohort ethics approvals

ALSPAC

Ethical approval was obtained from the ALSPAC Ethics and Law Committee and the Local Research Ethics Committees. Consent for biological samples has been collected in accordance with the Human Tissue Act (2004). Informed consent for the use of data collected via questionnaires and clinics was obtained from participants following the recommendations of the ALSPAC Ethics and Law Committee at the time (details and reference numbers of all ethics approvals can be found at (https://www.bristol.ac.uk/media-library/sites/alspac/documents/governance/Research_Ethics_Committee_approval_references.pdf).

BiB

Ethical approval for the study was granted by the Bradford National Health Service Research Ethics Committee (ref 06/Q1202/48), and all participants gave written informed consent. The ALL IN sub-study had ethical approval from the London School of Hygiene & Tropical Medicine ethics committee (ref: 5320) and the Bradford Research Ethics committee (ref: 08/H1302/21). Parents (usually the mother) gave informed, written consent to take part in the study.

MoBa

The current study is based on version 12 of the quality-assured data files released for research in 2019. The establishment of MoBa and initial data collection was based on a license from the Norwegian Data Protection Agency and approval from The Regional Committees for Medical and Health Research Ethics. The MoBa cohort is currently regulated by the Norwegian Health Registry Act. The current study was approved by The Regional Committees for Medical and Health Research Ethics of South/East Norway (ref 2018/1256).

UK Biobank

The UK Biobank has approval from the North West Multi-centre Research Ethics Committee (MREC) as a Research Tissue Bank (RTB) approval. This RTB approval was granted initially in 2011 (11/NW/0382) and it is renewed on a 5-yearly cycle, with the latest one successfully renewed in 2021 (21/NW/0157).

References

1. Richardson TG, Sanderson E, Elsworth B *et al.* Use of genetic variation to separate the effects of early and later life adiposity on disease risk: mendelian randomisation study, Research. *BMJ* 2020;**369**:m1203. https://doi.org/10.1136/bmj.m1203.

2. Brandkvist M, Bjørngaard JH, Ødegård RA *et al.* Separating the genetics of childhood and adult obesity: a validation study of genetic scores for body mass index in adolescence and adulthood in the HUNT Study. *Hum Mol Genet* 2021;**29**(24):3966–73. https://doi.org/10.1093/hmg/ddaa256.

3. Waterfield S, Richardson TG, Davey Smith G *et al.* Life course effects of genetic susceptibility to higher body size on body fat and lean mass: prospective cohort study. *International Journal of Epidemiology* 23 Mar. 2023:dyad029. https://doi.org/10.1093/ije/dyad029.

4. Winkler TW, Justice AE, Graff M *et al.* The Influence of Age and Sex on Genetic Associations with Adult Body Size and Shape: A Large-Scale Genome-Wide Interaction Study. *PLOS Genetics* 2015;**11**(10):e1005378. https://doi.org/10.1371/journal.pgen.1005378.

5. Pervjakova N, Moen GH, Borges MC *et al.* Multi-ancestry genome-wide association study of gestational diabetes mellitus highlights genetic links with type 2 diabetes. *Hum Mol Genet* 2022;**31**(19):3377–91. https://doi.org/10.1093/hmg/ddac050.

6. Steinthorsdottir V, McGinnis R, Williams NO *et al.* Genetic predisposition to hypertension is associated with preeclampsia in European and Central Asian women. *Nat Commun* 2020;**11**(1):5976. https://doi.org/10.1038/s41467-020-19733-6.

7. Solé-Navais P, Flatley C, Steinthorsdottir V *et al.* Genetic effects on the timing of parturition and links to fetal birth weight. *Nat Genet* 2023;**55**(4):559–67. https://doi.org/10.1038/s41588-023-01343-9.

8. Guintivano J, Byrne EM, Kiewa J *et al.* Meta-Analyses of Genome-Wide Association Studies for Postpartum Depression. *Am J Psychiatry* 2023;**180**(12):884–95. https://doi.org/10.1176/appi.ajp.20230053.

9. Willer CJ, Li Y, Abecasis GR. METAL: fast and efficient meta-analysis of genomewide association scans. *Bioinformatics* 2010;**26**(17):2190–1. https://doi.org/10.1093/bioinformatics/btq340.

10. McBride N, Clayton GL, Soares AG *et al.* Cohort profile: the Mendelian randomisation in pregnancy (MR-PREG) collaboration – improving evidence for prevention and treatment of adverse pregnancy and perinatal outcomes, Epidemiology. *BMJ Open* 2026;**16**(3):e103753. https://doi.org/10.1136/bmjopen-2025-103753.

11. Hughes RA, Heron J, Sterne JAC *et al.* Accounting for missing data in statistical analyses: multiple imputation is not always the answer. *International Journal of Epidemiology* 2019;**48**(4):1294–304. https://doi.org/10.1093/ije/dyz032.

12. Prince C, Sharp GC, Howe LD *et al.* The relationships between women's reproductive factors: a Mendelian randomisation analysis. *BMC Medicine* 2022;**20**(1):103. https://doi.org/10.1186/s12916-022-02293-5.

13. Bowden J, Davey Smith G, Burgess S. Mendelian randomization with invalid

instruments: effect estimation and bias detection through Egger regression. *International Journal of Epidemiology* 2015;**44**(2):512–25. https://doi.org/10.1093/ije/dyv080.

14. Sanderson E, Windmeijer F. A weak instrument F-Test in linear IV models with multiple endogenous variables. *Journal of Econometrics* Endogeneity Problems in Econometrics, 2016;**190**(2):212–21. https://doi.org/10.1016/j.jeconom.2015.06.004.

15. Sanderson E, Spiller W, Bowden J. Testing and correcting for weak and pleiotropic instruments in two-sample multivariable Mendelian randomization. *Stat Med* 2021;**40**(25):5434–52. https://doi.org/10.1002/sim.9133.

16. eaiton. *Eaiton/Menarche_pregnancy*. 12 May 2023, 24 Apr. 2024. https://github.com/eaiton/menarche_pregnancy (17 Mar. 2025, date last accessed).

17. Burgess S, Davies NM, Thompson SG. Bias due to participant overlap in two-sample Mendelian randomization. *Genet Epidemiol* 2016;**40**(7):597–608. https://doi.org/10.1002/gepi.21998.

18. Beaumont RN, Flatley C, Vaudel M *et al.* Genome-wide association study of placental weight identifies distinct and shared genetic influences between placental and fetal growth. *Nat Genet* 2023;**55**(11):1807–19. https://doi.org/10.1038/s41588-023-01520-w.

19. Wu Y, Zhong X, Lin Y *et al.* Estimating genetic nurture with summary statistics of multigenerational genome-wide association studies. *Proceedings of the National Academy of Sciences* 2021;**118**(25):e2023184118. https://doi.org/10.1073/pnas.2023184118.

20. Kentistou KA, Kaisinger LR, Stankovic S *et al.* Understanding the genetic complexity of puberty timing across the allele frequency spectrum. *Nat Genet* 2024;**56**(7):1397–411. https://doi.org/10.1038/s41588-024-01798-4.
