# Supplementary material for: Age at menarche and adverse pregnancy and perinatal outcomes: triangulating evidence from multivariable and Mendelian randomization analyses
Source: Int J Epidemiol. 2026 Jun 24;55(4):dyag094. doi: 10.1093/ije/dyag094 (PMC13291527; doi:10.1093/ije/dyag094)
Supplement: dyag094_Supplementary_Data [file dyag094_supplementary_data.zip › ije-2025-09-1838-File007.pdf]

## Supplementary Material: figures and tables

Table of Contents

Figure S1. Flow diagram describing instrumental variable selection for main univariable Mendelian randomization analysis. ..... 3

Figure S2. Flow diagram describing instrumental variable selection for multivariable Mendelian randomization (MVMR) analysis. ..... 4

Figure S3. Flow diagram describing ALSPAC participant inclusion in observational multivariable regression analysis ..... 5

Figure S4. Observational multivariable regression and Mendelian randomization estimates for the effect of a one-year increase in age at menarche on birthweight (in SD, ~ 601.9 grams). ..... 6

Figure S5. Observational multivariable regression and Mendelian randomization estimates for the effect of a one-year increase in age at menarche on perinatal depression. ..... 7

Figure S6. Observational multivariable regression estimates for the effect of early or late age at menarche on binary outcomes. ..... 8

Figure S7. Observational multivariable regression estimates for the effect of early or late age at menarche on birthweight (in SD, ~ 601.9 grams). ..... 9

Figure S8. Observational multivariable regression estimates for the effect of a one-year increase in age at menarche on binary outcomes, for models excluding potential colliders (age at delivery and parity). ..... 10

Figure S9. Observational multivariable regression estimates for the effect of a one-year increase in age at menarche on birthweight (in SD, ~ 601.9 grams), for models excluding potential colliders (age at delivery and parity). ..... 11

Figure S10. Leave-one-study-out univariate Mendelian randomization estimates for binary outcomes. ..... 12

Figure S11. Leave-one-study-out multivariable Mendelian randomization estimates for binary outcomes ..... 13

Figure S12. Fetal genotype adjusted univariate Mendelian randomization effect estimates for binary outcomes ..... 14

Figure S13. Fetal genotype adjusted multivariable Mendelian randomization effect estimates for binary outcomes ..... 15

Figure S14. Mendelian randomization estimates using genetic instruments with known biological roles in pubertal timing on binary outcomes. ..... 16

Figure S15. Mendelian randomization estimates using genetic instruments with known biological roles in pubertal timing on offspring birthweight (in SD, ~ 601.9 grams). ..... 17

Figure S16. Correlation between SNP effects in combined and female only sample, for genetic variants instrumenting adiposity. ..... 18

Figure S17. Relation of age at menarche with adjusted odds of binary outcomes. .. 20

Figure S18. Relation of maternal age at menarche with offspring birthweight (in SD, ~ 601.9 grams). ........ 21
Table S1. Description of GWAS used in Mendelian randomization analyses. ........ 22
Table S2. Pregnancy and perinatal outcome definitions for MR-PREG collaboration. ........ 23
Table S3A. Sample sizes contributing to binary adverse pregnancy and perinatal outcomes with maternal genetic data, across MR-PREG collaboration cohorts and publicly available GWAS. ........ 26
Table S3B. Sample sizes contributing to continuous adverse pregnancy and perinatal outcomes with maternal genetic data, across MR-PREG collaboration cohorts and publicly available GWAS. ........ 27
Table S4. Description of variables used in ALSPAC observational multivariable regression models ........ 28
Table S5. Outcome sample sizes within ALSPAC sample. ........ 29
Table S6. ALSPAC participant baseline characteristics by age at menarche[1] ........ 30
Table S7. MR-Egger intercept values testing for evidence of directional pleiotropy for estimated effects of age at menarche on all outcomes. ........ 32
Table S8. Cochran's Q-statistics testing for evidence of between-SNP heterogeneity of effects for MR IVW and multivariable MR (MVMR) estimated effects of age at menarche on all outcomes. ........ 33
Table S9. Sample overlap between age at menarche exposure GWAS (n=632,955) and meta-analysed outcome GWAS, by outcome. ........ 34
Table S10. Sample overlap between pre-pubertal body size exposure GWAS (n=453,169) and meta-analysed outcome GWAS, by outcome. ........ 35
Table S11. Leave-one-study-out Mendelian randomization estimates for birthweight ........ 36
Table S12. Fetal genotype adjusted Mendelian randomization estimates for birthweight ........ 37
Table S13. Cochran's Q-statistics testing for evidence of between-SNP heterogeneity of effects for MR IVW and multivariable MR (MVMR) estimated effects of age at menarche on all outcomes, after accounting for fetal genetic effects. ........ 38
Table S14. Genetic variants with known biological roles in pubertal timing, used as instrument for sensitivity analysis. ........ 39
Table S15. Likelihood ratio tests comparing nested models of early, intermediate, and late categories of age at menarche for each outcome.[1] ........ 40

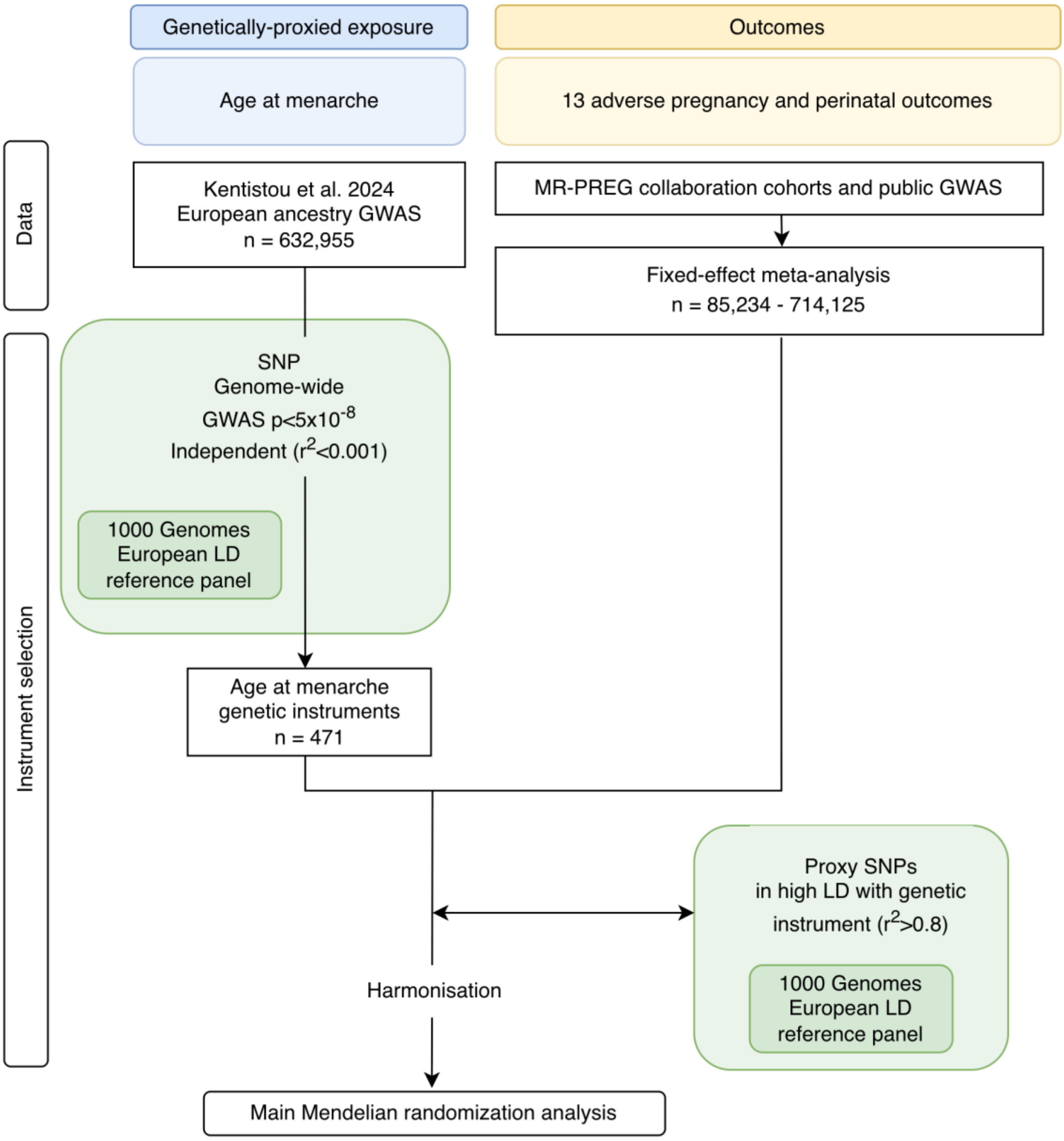


**Figure S1. Flow diagram describing instrumental variable selection for main univariable Mendelian randomization analysis.**
SNP = single nucleotide polymorphism, GWAS = genome-wide association study, LD = linkage disequilibrium. Generated using draw.io (https://github.com/jgraph/drawio).

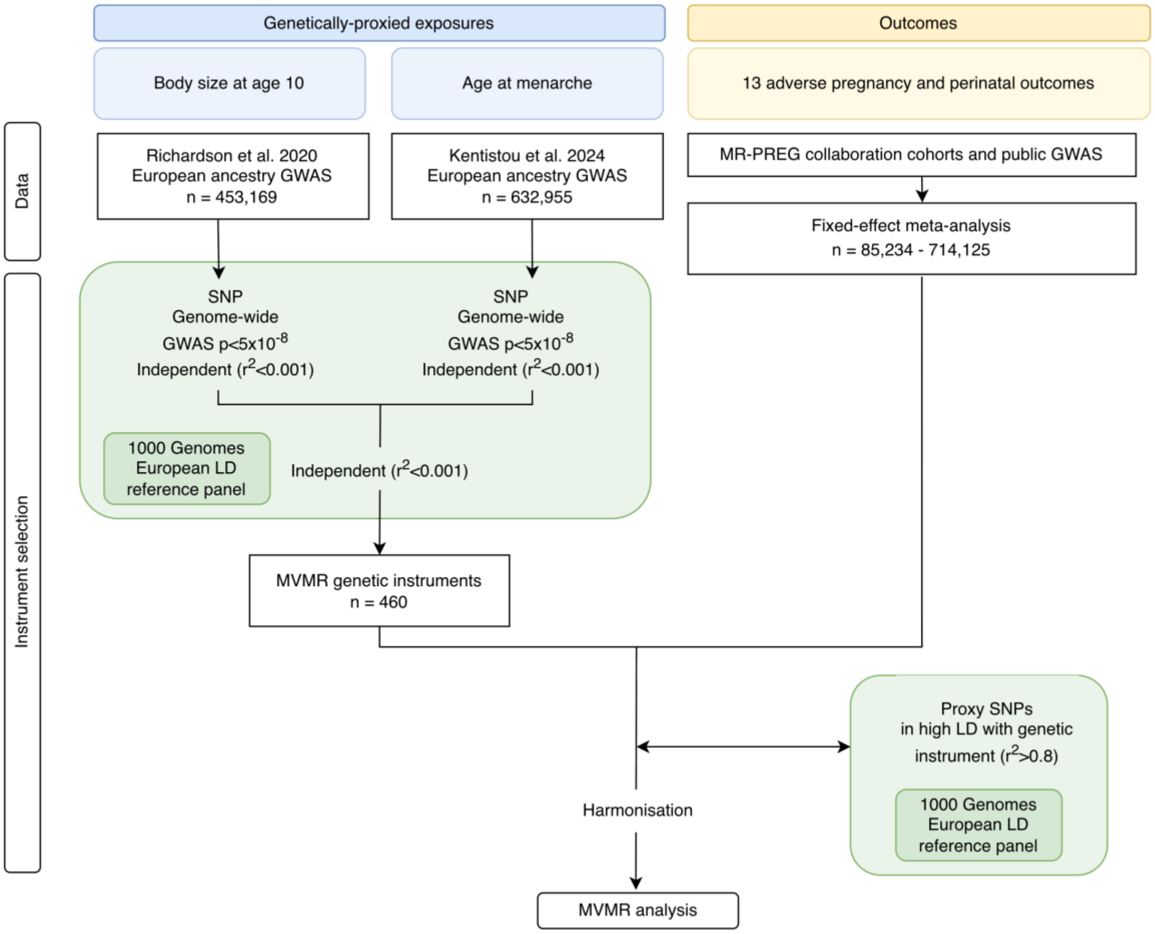


**Figure S2. Flow diagram describing instrumental variable selection for multivariable Mendelian randomization (MVMR) analysis.**
After selecting independent instruments for both age at menarche and body size at age 10, the resulting SNPs were clumped again to select an independent set of instruments. SNP = single nucleotide polymorphism, GWAS = genome-wide association study, LD = linkage disequilibrium. Generated using draw.io (https://github.com/jgraph/drawio).

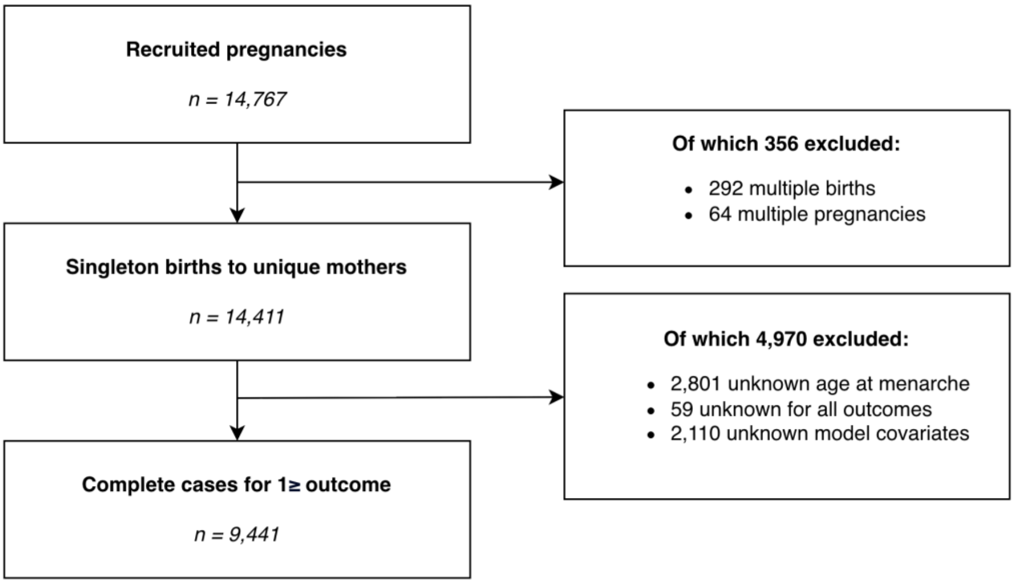


**Figure S3. Flow diagram describing ALSPAC participant inclusion in observational multivariable regression analysis.**
Multiple pregnancies refers to instances where the same mother was included in the cohort for two separate pregnancies, while multiple births refers to twins or triplets. Generated using draw.io (https://github.com/jgraph/drawio).

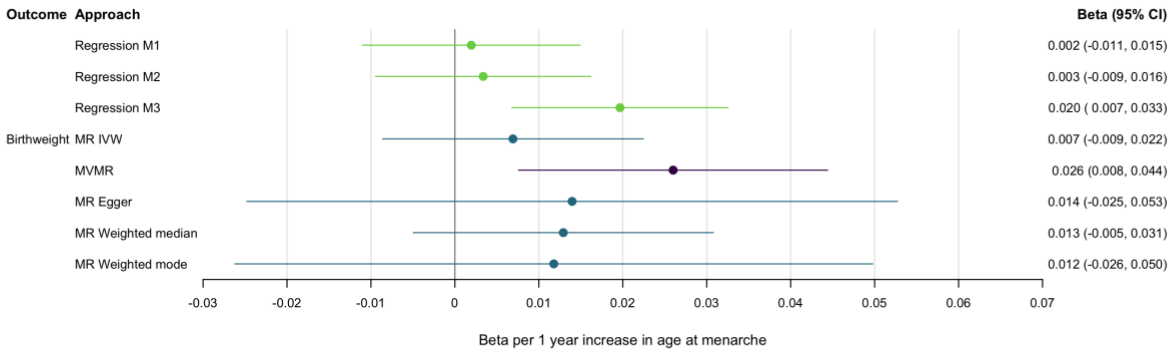


**Figure S4. Observational multivariable regression and Mendelian randomization estimates for the effect of a one-year increase in age at menarche on birthweight (in SD, ~ 601.9 grams).**
Illustrating i) multivariable regression model 1 (M1): unadjusted estimate, ii) multivariable regression model 2 (M2): estimate adjusted for highest educational attainment, ethnicity, age at delivery, parity, and offspring sex, iii) multivariable regression estimate model 3 (M3): model 2 estimate with additional adjustment for adiposity, iv) inverse variance weighted (IVW) Mendelian randomization estimate, v) multivariable Mendelian randomization (MVMR) estimate accounting for adiposity, vi) MR Egger Mendelian randomization estimate, vii) weighted median Mendelian randomization estimate, viii) weighted mode Mendelian randomization estimate. Observational multivariable regression estimates are shown in green, univariable MR estimates in blue, and MVMR estimates in purple.

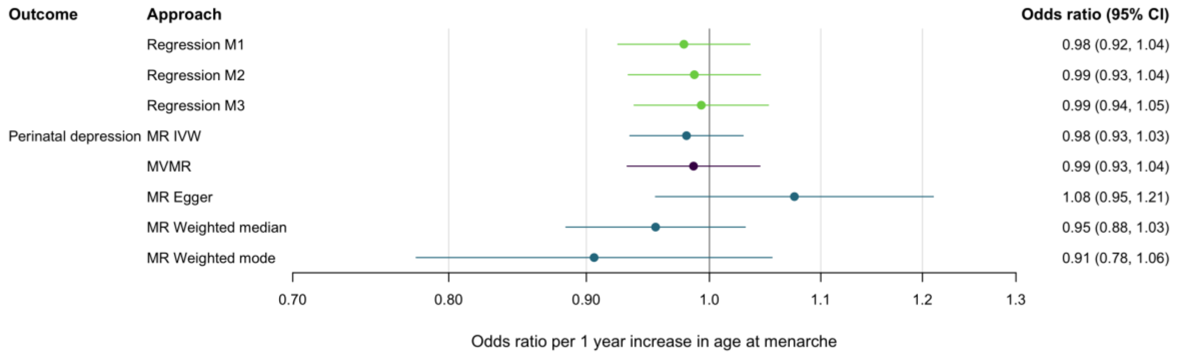


**Figure S5. Observational multivariable regression and Mendelian randomization estimates for the effect of a one-year increase in age at menarche on perinatal depression.**
Illustrating i) multivariable regression model 1 (M1): unadjusted estimate, ii) multivariable regression model 2 (M2): estimate adjusted for highest educational attainment, ethnicity, age at delivery, parity, and offspring sex, iii) multivariable regression estimate model 3 (M3): model 2 estimate with additional adjustment for adiposity, iv) inverse variance weighted (IVW) Mendelian randomization estimate, v) multivariable Mendelian randomization (MVMR) estimate accounting for adiposity, vi) MR Egger Mendelian randomization estimate, vii) weighted median Mendelian randomization estimate, viii) weighted mode Mendelian randomization estimate. Observational multivariable regression estimates are shown in green, univariable MR estimates in blue, and MVMR estimates in purple.

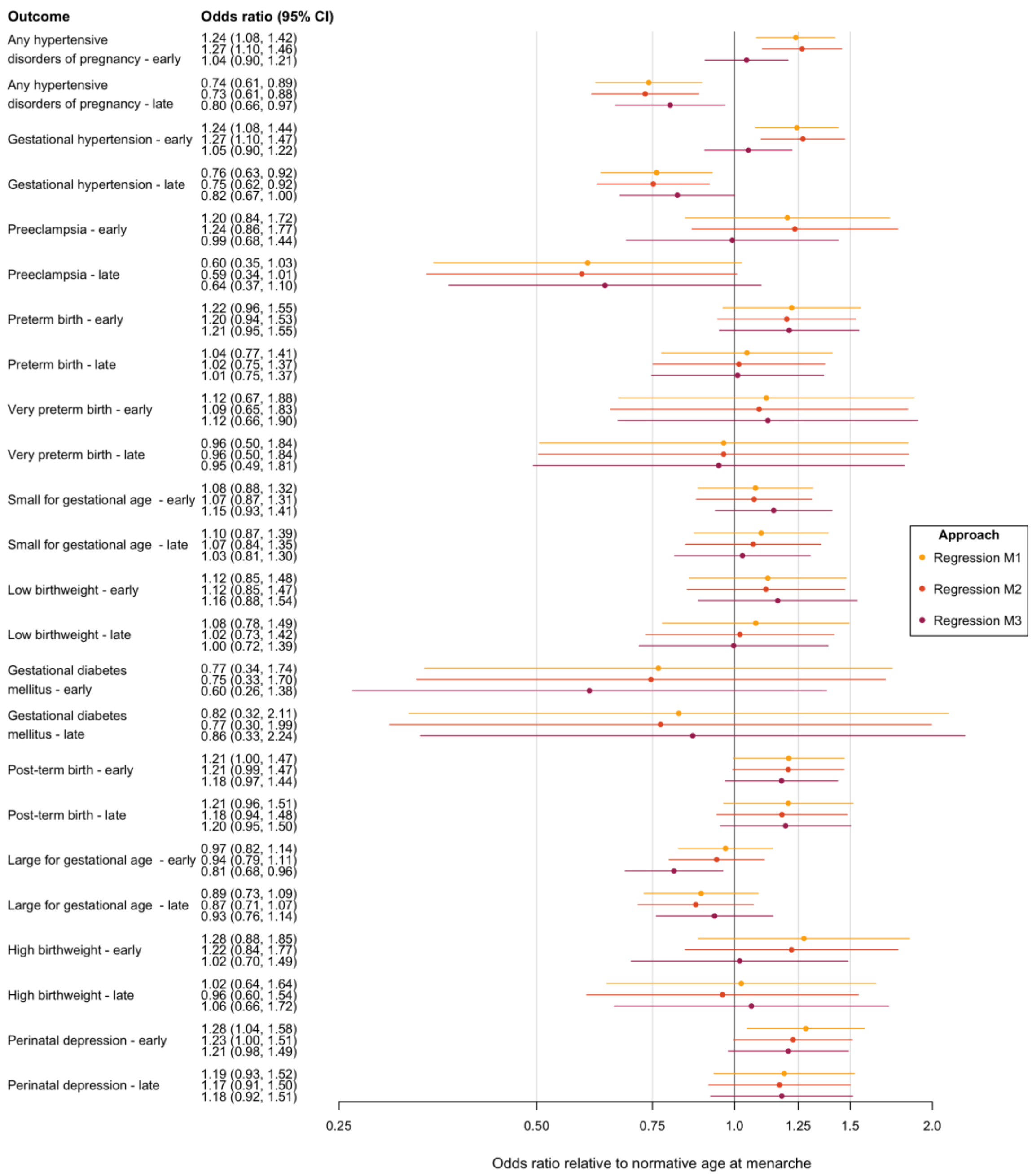


**Figure S6. Observational multivariable regression estimates for the effect of early or late age at menarche on binary outcomes.**

Effect estimates compared to intermediate age at menarche as the reference category. Early age at menarche was defined as less than one standard deviation below the mean (11 and younger), intermediate within one standard deviation of the mean (12 to 14 inclusive), and late age at menarche as more than one standard deviation above the mean (15 and older). Illustrating i) multivariable regression model 1 (M1): unadjusted estimate, ii) multivariable regression model 2 (M2): estimate adjusted for highest educational attainment, ethnicity, age at delivery, parity, and offspring sex, iii) multivariable regression estimate model 3 (M3): model 2 estimate with additional adjustment for adiposity.

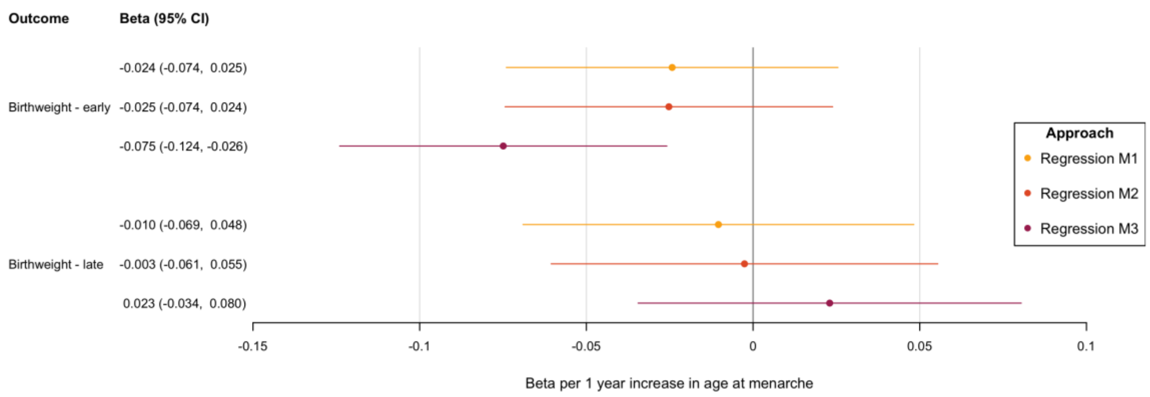


**Figure S7. Observational multivariable regression estimates for the effect of early or late age at menarche on birthweight (in SD, ~ 601.9 grams).**
Effect estimates compared to intermediate age at menarche as the reference category. Early age at menarche was defined as less than one standard deviation below the mean (11 and younger), intermediate within one standard deviation of the mean (12 to 14 inclusive), and late age at menarche as more than one standard deviation above the mean (15 and older). Illustrating i) multivariable regression model 1 (M1): unadjusted estimate, ii) multivariable regression model 2 (M2): estimate adjusted for highest educational attainment, ethnicity, age at delivery, parity, and offspring sex, iii) multivariable regression estimate model 3 (M3): model 2 estimate with additional adjustment for adiposity.

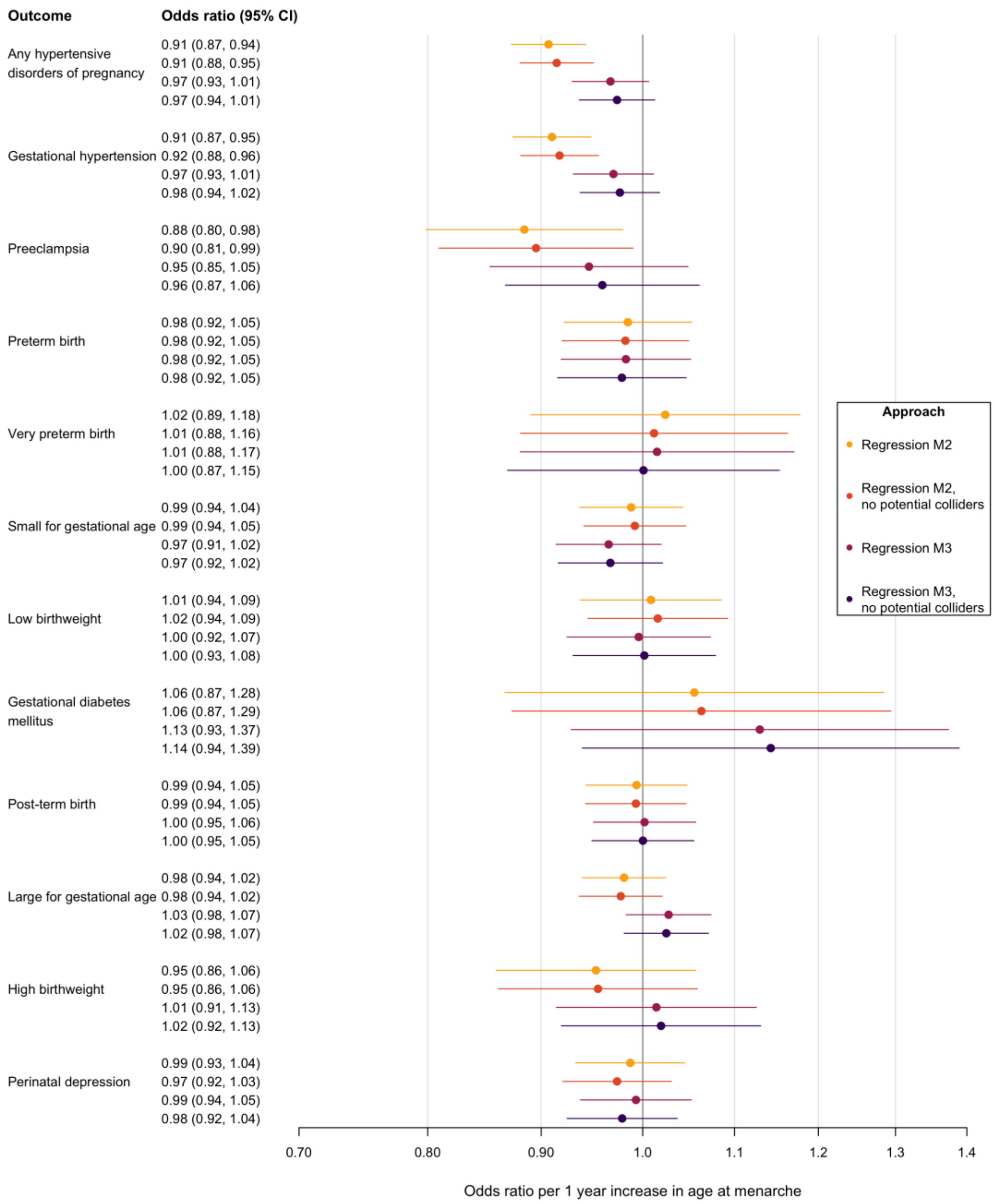


**Figure S8. Observational multivariable regression estimates for the effect of a one-year increase in age at menarche on binary outcomes, for models excluding potential colliders (age at delivery and parity).**
Illustrating i) multivariable regression model 2: estimate adjusted for highest educational attainment, ethnicity, age at delivery, parity, and offspring sex, i) multivariable regression model 2 excluding potential colliders: estimate adjusted for highest educational attainment, ethnicity, and offspring sex, iii) multivariable regression estimate model 3 (M3): model 2 estimate with additional adjustment for adiposity, iv) multivariable regression estimate model 3 excluding potential colliders: estimate adjusted for highest educational attainment, ethnicity, and offspring sex, with additional adjustment for adiposity.

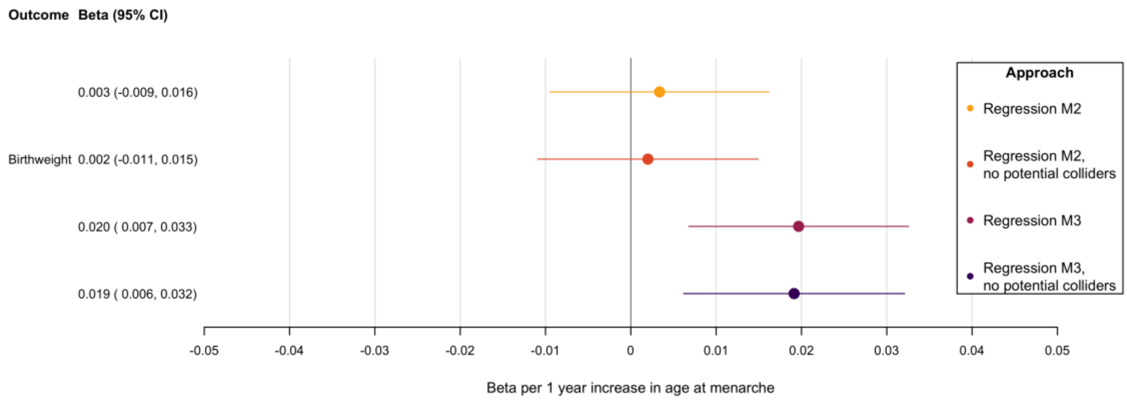


**Figure S9. Observational multivariable regression estimates for the effect of a one-year increase in age at menarche on birthweight (in SD, ~ 601.9 grams), for models excluding potential colliders (age at delivery and parity).**
Illustrating i) multivariable regression model 2: estimate adjusted for highest educational attainment, ethnicity, age at delivery, parity, and offspring sex, i) multivariable regression model 2 excluding potential colliders: estimate adjusted for highest educational attainment, ethnicity, and offspring sex, iii) multivariable regression estimate model 3 (M3): model 2 estimate with additional adjustment for adiposity, iv) multivariable regression estimate model 3 excluding potential colliders: estimate adjusted for highest educational attainment, ethnicity, and offspring sex, with additional adjustment for adiposity.

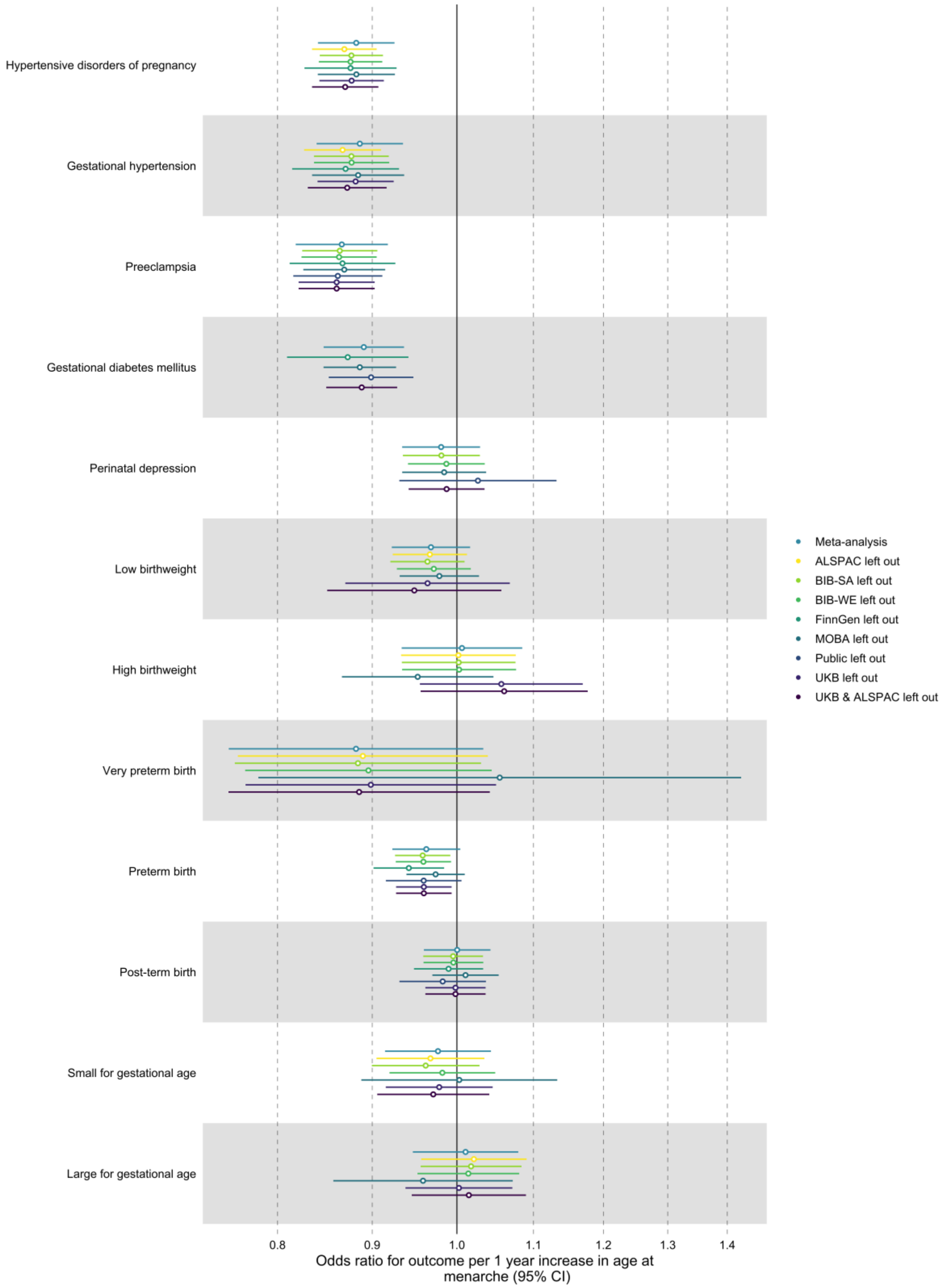


**Figure S10. Leave-one-study-out univariate Mendelian randomization estimates for binary outcomes.**
Analyses leaving out both UK Biobank (UKB) & ALSPAC aim to address study overlap, since these are the overlapping studies between exposure and outcome GWAS.

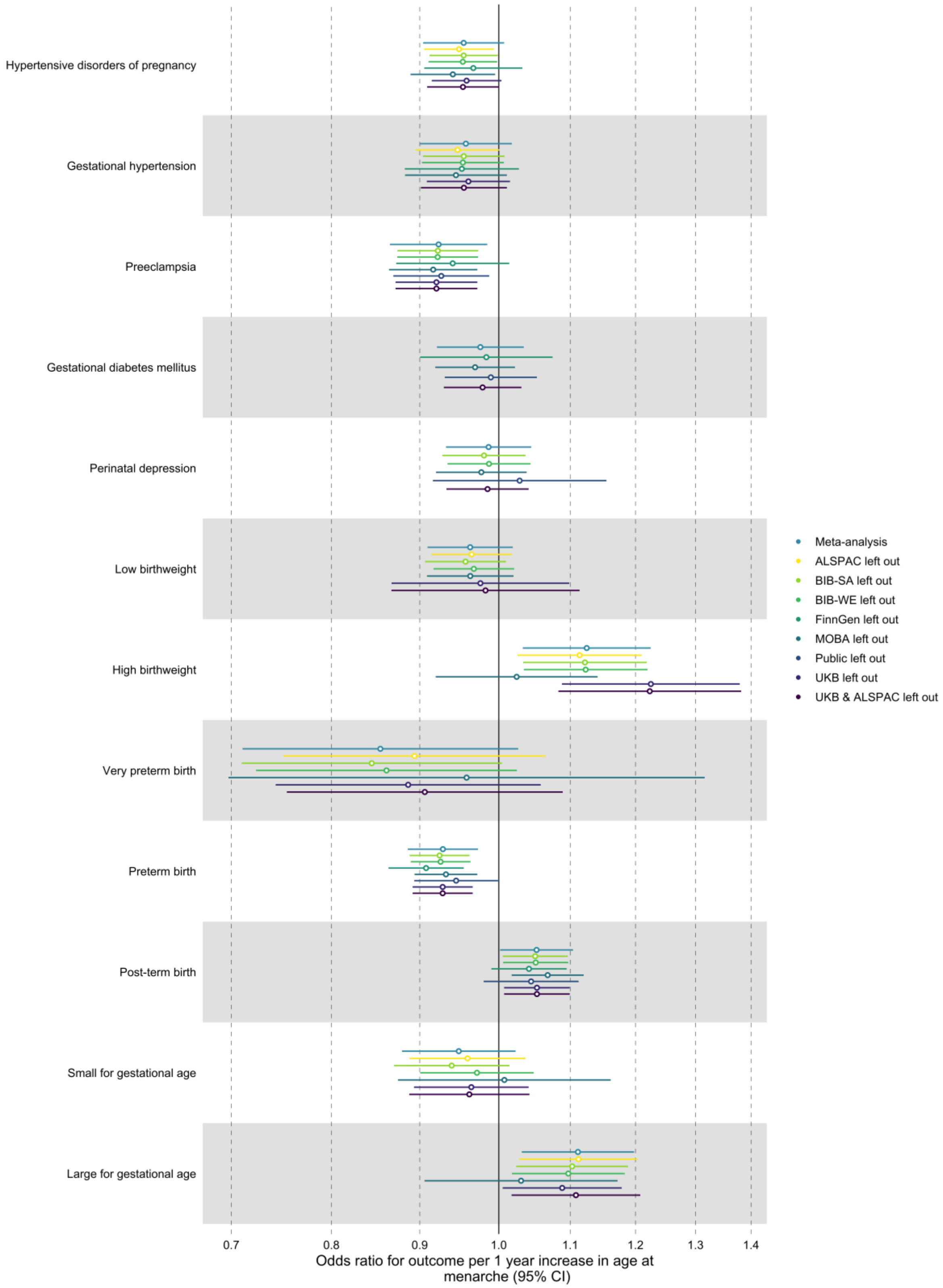


**Figure S11. Leave-one-study-out multivariable Mendelian randomization estimates for binary outcomes**
Analyses leaving out both UK Biobank (UKB) & ALSPAC aim to address study overlap, since these are the overlapping studies between exposure and outcome GWAS.

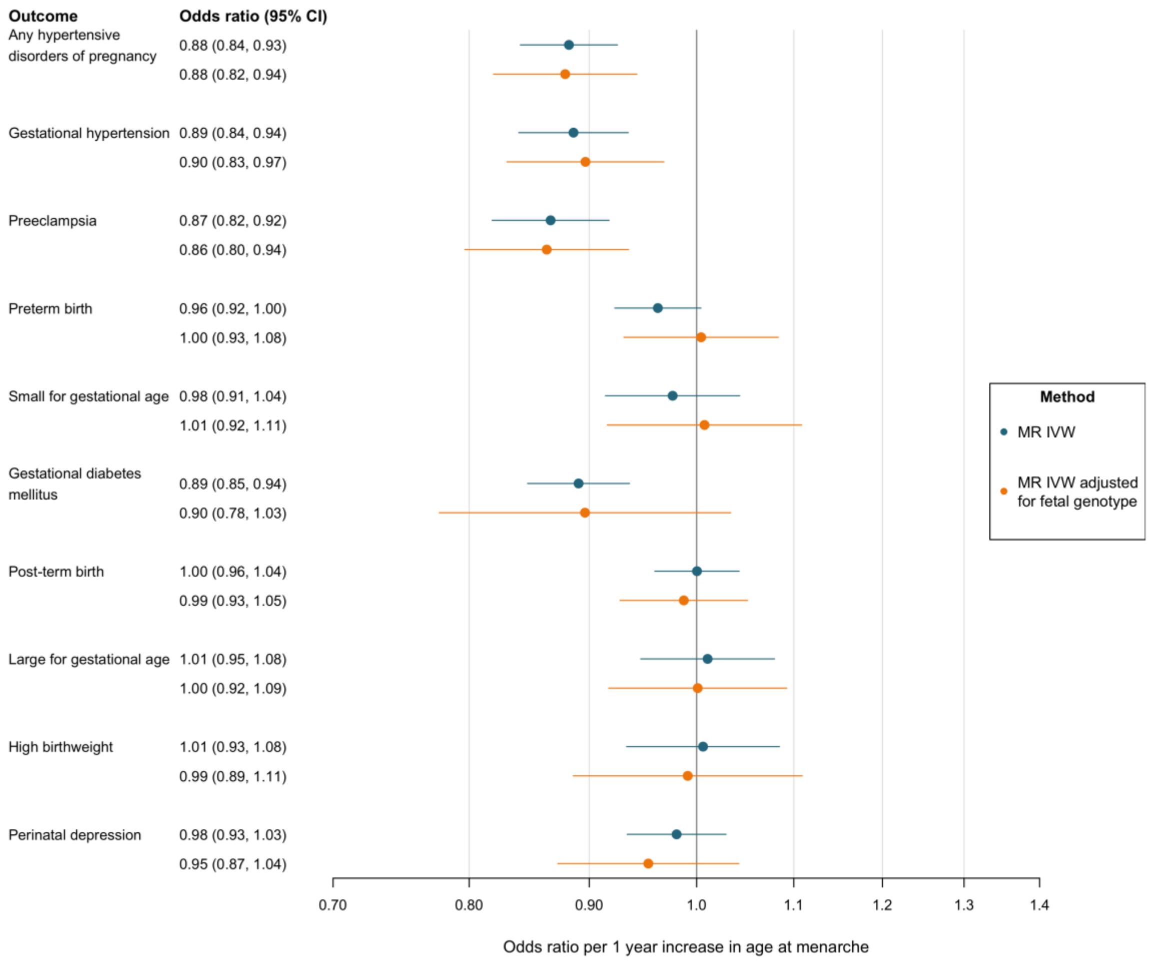


**Figure S12. Fetal genotype adjusted univariate Mendelian randomization effect estimates for binary outcomes**

Effect estimates for a one-year increase in age at menarche on all binary outcomes, comparing MR IVW unadjusted estimate (i.e. main results) against MR IVW estimate adjusted for fetal genotype. IVW = inverse variance weighted.

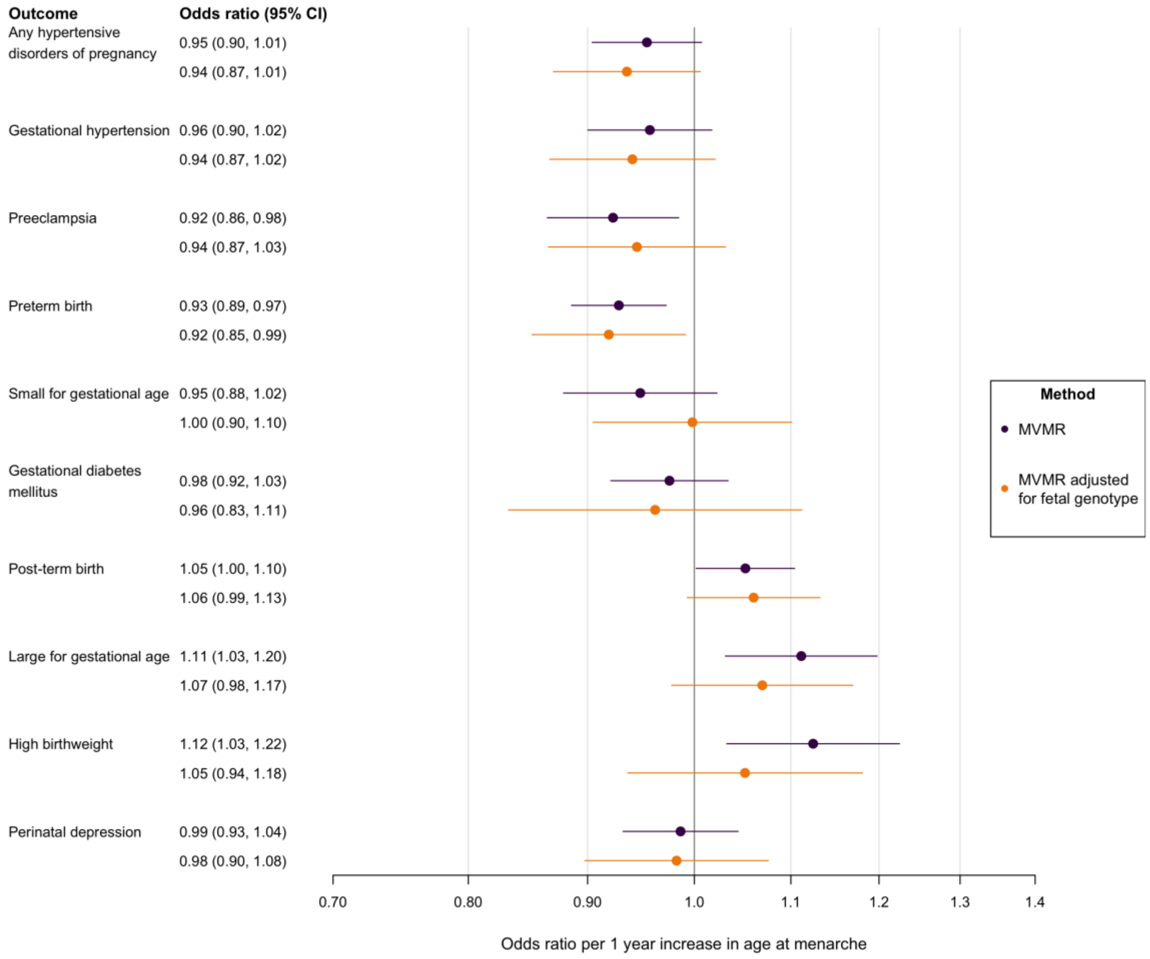


**Figure S13. Fetal genotype adjusted multivariable Mendelian randomization effect estimates for binary outcomes**

Effect estimates for a one-year increase in age at menarche on all binary outcomes, comparing MVMR unadjusted estimate against MVMR estimate adjusted for fetal genotype.

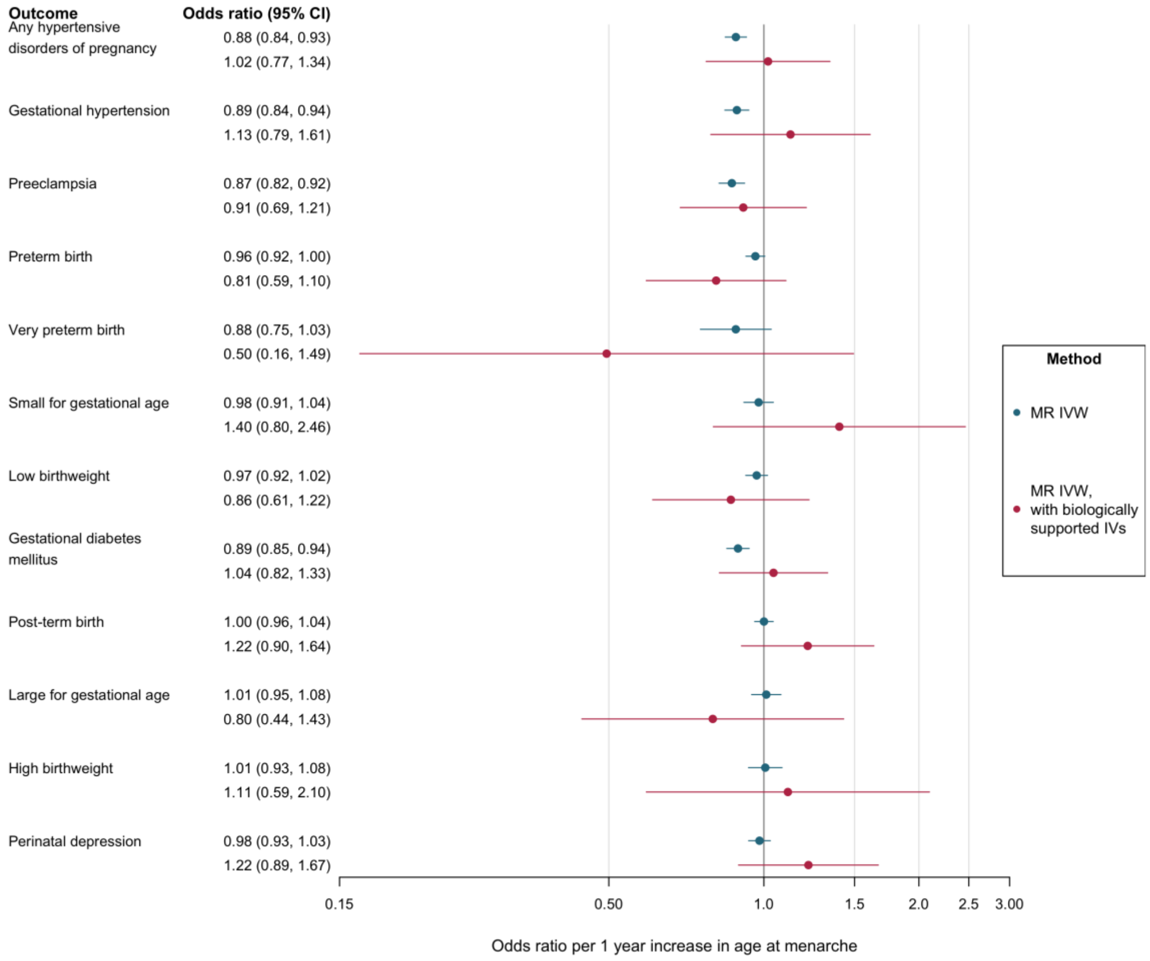


**Figure S14. Mendelian randomization estimates using genetic instruments with known biological roles in pubertal timing on binary outcomes.**
Effect estimates for a one-year increase in age at menarche on all binary outcomes, comparing MR IVW against MR IVW using only biologically-supported instrumental variables. IV = instrumental variable. IVW = inverse variance weighted.

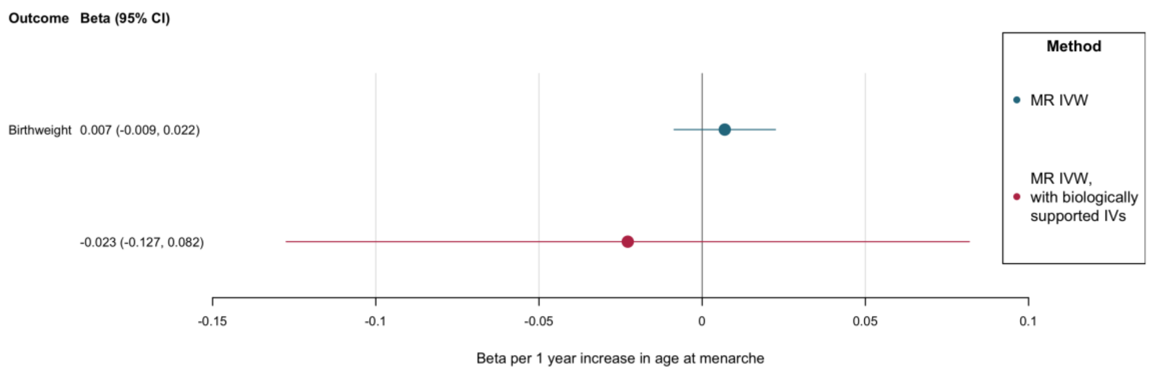


**Figure S15. Mendelian randomization estimates using genetic instruments with known biological roles in pubertal timing on offspring birthweight (in SD, ~ 601.9 grams).**
Effect estimates for a one-year increase in age at menarche on all binary outcomes, comparing MR IVW against MR IVW using only biologically-supported instrumental variables. IV = instrumental variable. IVW = inverse variance weighted.

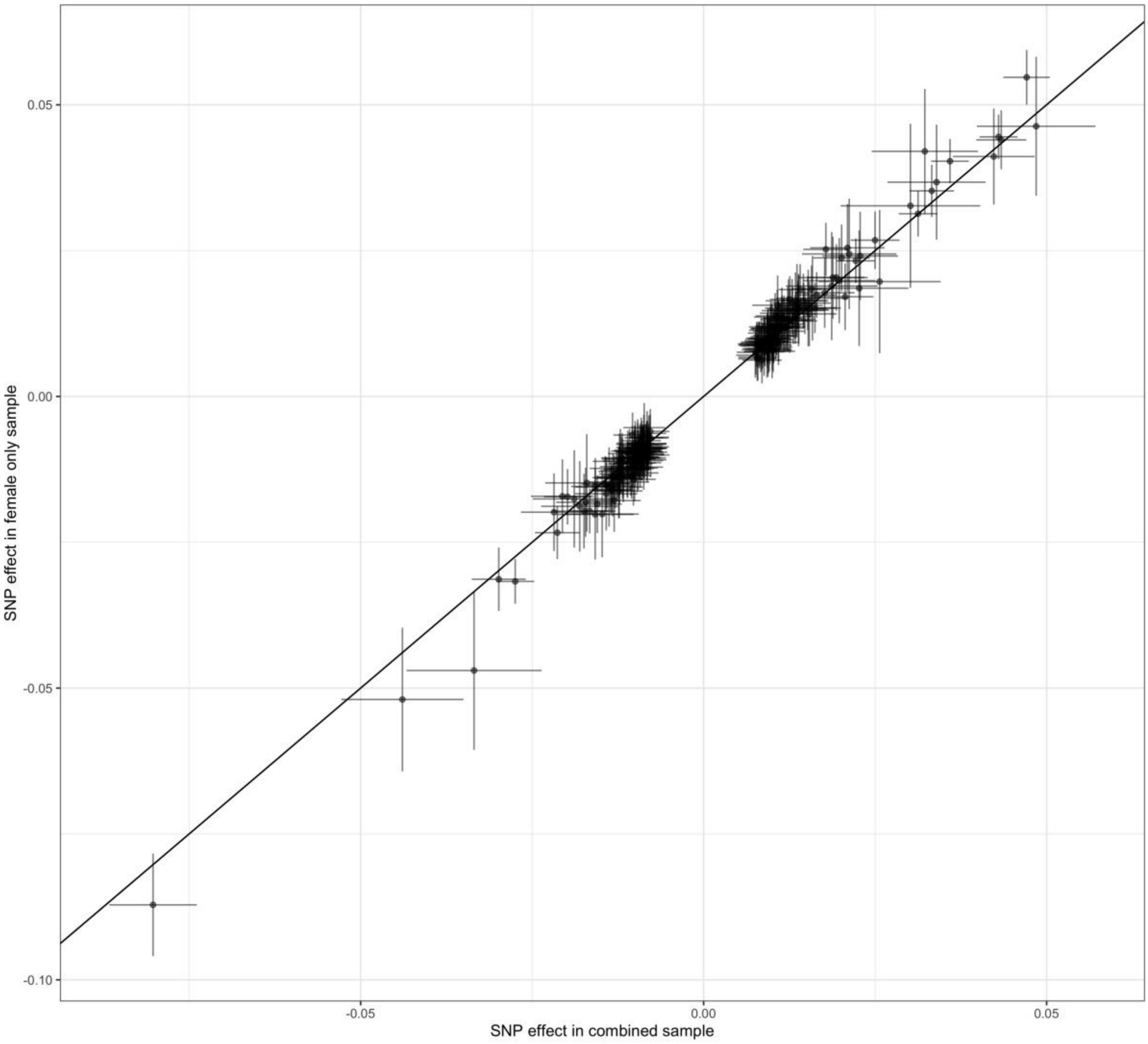


**Figure S16. Correlation between SNP effects in combined and female only sample, for genetic variants instrumenting adiposity.**
Alleles harmonised and all effects shown with 95% confidence intervals. The overlapping female-specific (n=246,511) and combined samples (n=453,169) are from the UK Biobank. Effect sizes reflect additive change in body size category; for further details see Richardson *et al.* 2020.

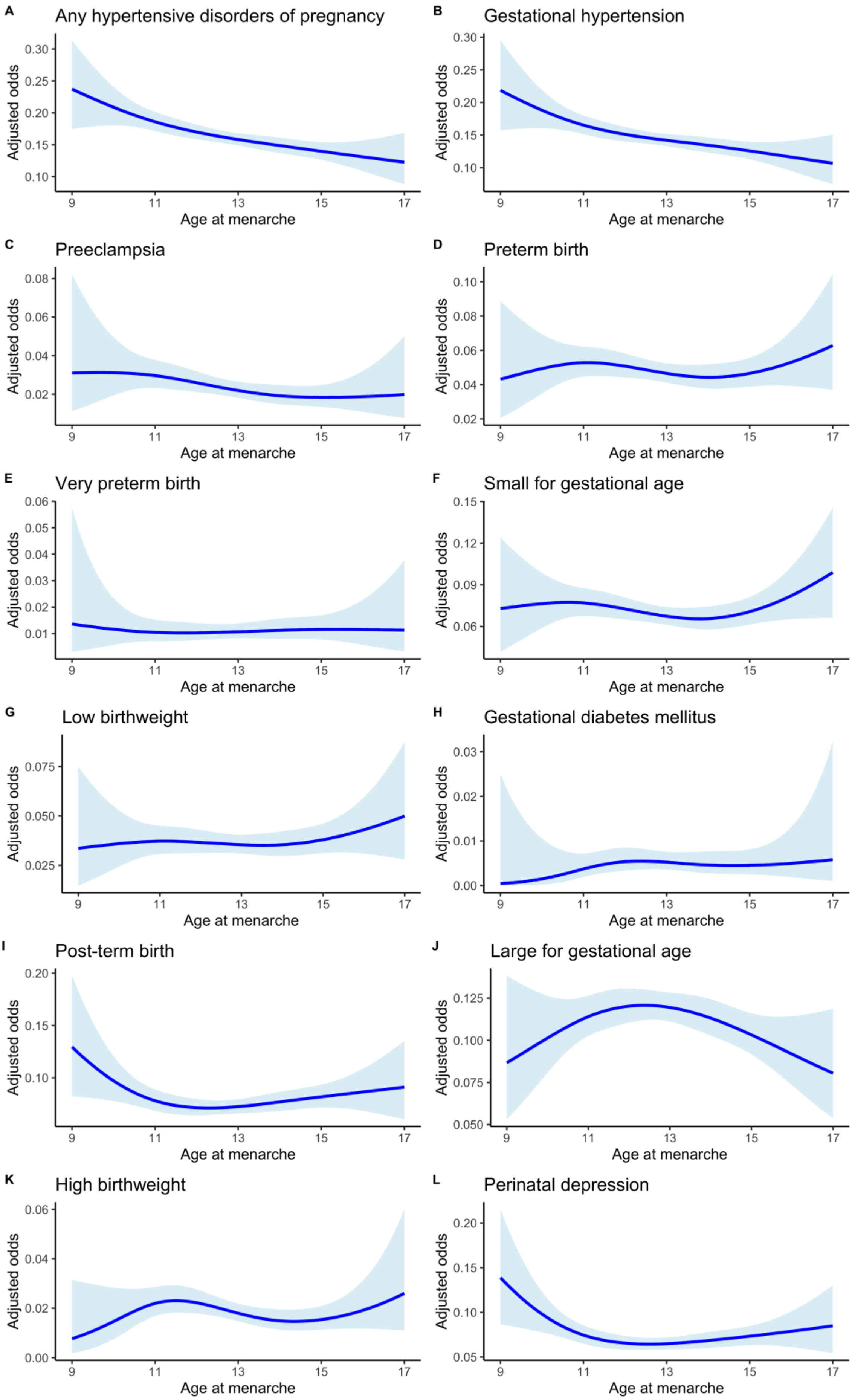
A
Any hypertensive disorders of pregnancy
B
Gestational hypertension
C
Preeclampsia
D
Preterm birth
E
Very preterm birth
F
Small for gestational age
G
Low birthweight
H
Gestational diabetes mellitus
I
Post-term birth
J
Large for gestational age
K
High birthweight
L
Perinatal depression
Adjusted odds
Age at menarche

**Figure S17. Relation of age at menarche with adjusted odds of binary outcomes.**
Restricted cubic spline models adjusted for highest educational attainment, ethnicity, age at delivery, parity, offspring sex, and pre-pregnancy BMI. Light blue shaded area illustrates 95% confidence intervals.

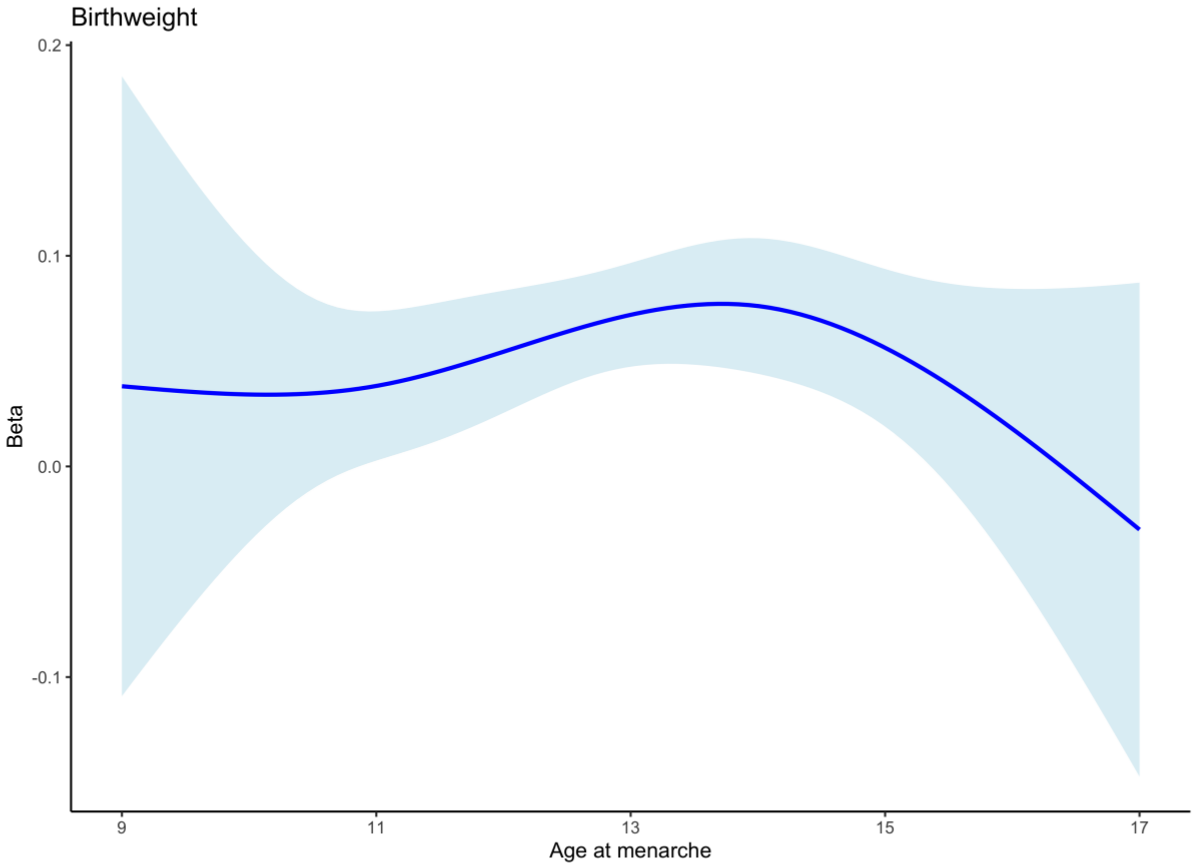


**Figure S18. Relation of maternal age at menarche with offspring birthweight (in SD, ~ 601.9 grams).**
Restricted cubic spline model adjusted for highest educational attainment, ethnicity, age at delivery, parity, offspring sex, and pre-pregnancy BMI. Light blue shaded area illustrates 95% confidence intervals.

**Table S1. Description of GWAS used in Mendelian randomization analyses.**

| Trait | Author and year | GWAS model covariates | Ancestry adjustment | Sample size |
|---|---|---|---|---|
| Age at menarche | Kentistou et al 2024 | Age | Principal components | 632,955 |
| Early life body size | Richardson et al 2020 | Age at baseline, genotyping array | Linear mixed model | 453,169 |
| MR-PREG pregnancy outcome GWAS | Various | Genotyping array<br><br>Firth correction used to account for case-control imbalance for binary outcomes. | Principal components and linear mixed models | 77,683 to 707,797 |

**Table S2. Pregnancy and perinatal outcome definitions for MR-PREG collaboration.**
[1]Contributing studies: ALSPAC (A), BiB (B), MoBa (M), UK Biobank (U), FinnGen (F), multiple publicly available GWAS (G). *Definitions of cases and controls in the publicly available GWAS might vary across studies contributing to the GWAS meta-analyses. **For birthweight, Z-scores were standardised against UK-WHO using Stata Zanthro package, and all estimates were additionally adjusted for gestational age.

| Outcomes | Contributing studies[1] | | | | | | Case definition | Control definition | Exclusions | | | |
|---|---|---|---|---|---|---|---|---|---|---|---|---|
| | A | B | M | U | F | G* | | | Multiple births | Duplicated pregnancies | Non-live births | Other |
| Gestational hypertension | x | x | x | | | | SBP ≥ 140 mmHg or DBP ≥ 90 mmHg on at least 2 occasions after 20 gestational weeks, with no concurrent proteinuria | No hypertensive disorders of pregnancy | x | x | x | Pre-existing hypertension |
| | | | | x | x | | ICD-10 code: O13 | | | | | |
| Pre-eclampsia | x | x | x | | | x | Gestational hypertension with concurrent proteinuria | | x | x | x | |
| | | | | x | x | x | ICD-10 code: O14 | | | | | |
| Hypertensive disorders of pregnancy | x | x | x | | | | Gestational hypertension or preeclampsia | | x | x | x | |
| | | | | x | x | | ICD-10 codes: O13, O14 | | | | | |
| Gestational diabetes mellitus | | | x | | | x | Self-reported diabetes arising in pregnancy | No gestational diabetes | x | x | x | Pre-existing diabetes |
| | x | | | | | x | Clinical diagnoses abstracted from medical records by research midwives | | | | | |
| | | | | x | x | x | ICD-10 code: O24 | | | | | |
| | | x | | | | x | Fasting glucose ≥ 6.1 mmol/L or 2 h post-load glucose ≥ 7.8 mmol/L in OGTT | | | | | |
| Perinatal depression | x | | | | | x | Mean EPDS total score across 12 and 32 gestational weeks and 8 weeks and 8 months postpartum > 12 | No depression | x | x | x | Pre-existing depression |
| | | x | | | | x | Mean score of questions 22 to 28 in the GHQ administered at cohort registration during pregnancy > the 85th percentile | | | | | |
| | | | | x | | x | Self-reported antenatal depression | | | | | |
| | | | x | | | | Mean of selective items from the (Hopkins) Symptoms Checklist-25 at 14 and 30 weeks (and 6 | | | | | |

| | | | | | | | | | | | |
|---|---|---|---|---|---|---|---|---|---|---|---|
| | | | | | | months post partum)>=2 | | | | | |
| Miscarriage | x | | x | x | | Miscarriage in the index or previous pregnancies; miscarriage in the index pregnancy was defined as fetal loss before 20 weeks gestation; miscarriage history in previous pregnancies was self-reported in questionnaires | No pregnancy loss | x | x | | |
| | | x | | x | x | Miscarriage in the index or previous pregnancies; information on miscarriage was abstracted from medical records | | | | | |
| Stillbirth | x | | | x | | Stillbirth in the index or previous pregnancies; stillbirth in the index pregnancy was defined as fetal death before delivery at 20 gestational weeks or more; stillbirth history in previous pregnancies was self-reported in questionnaires | | x | x | | |
| | | x | x | | | Stillbirth in the index or previous pregnancies; information on stillbirth was abstracted from medical records | | | | | |
| Very preterm birth | x | x | x | x | | Gestational age at birth < 34 weeks | Gestational age at birth ≥ 37 to < 42 weeks | x | x | x | |
| Preterm birth | x | x | x | x | | Gestational age at birth < 37 weeks | | x | x | x | |
| Post-term birth | x | x | x | x | | Gestational age at birth ≥ 42 weeks | | x | x | x | |
| Low birth weight | x | x | x | x | | Birth weight < 2500 g | Birth weight ≥ 2500 g to ≤ 4500 g | x | x | x | |
| High birth weight | x | x | x | x | | Birth weight > 4500 g | | x | x | x | |
| Small for gestational age (SGA) | x | x | x | x | | Birth weight-for-gestational age < 10th percentile | No SGA | x | x | x | |
| Large for gestational age (LGA) | x | x | x | x | | Birth weight-for-gestational age > 90th percentile | No LGA | x | x | x | |
| Birthweight | x | x | x | | | Abstracted from medical records | Standard deviation units (Z-score transformed separately in males and | x | x | x | Birth weight > 5 SD |

| | | | | |
|---|---|---|---|---|
| | | | females)** | |
| | x | Self-reported in questionnaires | | |

**Table S3A. Sample sizes contributing to binary adverse pregnancy and perinatal outcomes with maternal genetic data, across MR-PREG collaboration cohorts and publicly available GWAS.**

| Outcomes | Overall | | ALSPAC | | BiB | | MoBa | | UK Biobank | | FinnGen | | GWAS | |
|---|---|---|---|---|---|---|---|---|---|---|---|---|---|---|
| | Cases | Controls | Cases | Controls | Cases | Controls | Cases | Controls | Cases | Controls | Cases | Controls | Cases | Controls |
| Hypertensive disorders of pregnancy | 32,549 | 509,219 | 1,187 | 5,851 | 683 | 5,965 | 9,472 | 64,878 | 802 | 170,866 | 20,405 | 261,659 | | |
| Gestational hypertension | 20,777 | 507,155 | 1,029 | 5,851 | 499 | 5,965 | 6,681 | 64,877 | 518 | 171,149 | 12,050 | 259,313 | | |
| Pre-eclampsia | 19,408 | 652,584 | | | 184 | 5,965 | 2,628 | 60,335 | 354 | 171,311 | 9,023 | 259,313 | 7,219 | 155,660 |
| Gestational diabetes | 24,641 | 683,156 | | | | | 602 | 73,507 | | | 18,581 | 263,483 | 5,458 | 346,166 |
| Depression | 14,591 | 91,065 | | | 839 | 5,312 | 2,889 | 52,282 | | | | | 10,863 | 33,471 |
| Miscarriage | 89,086 | 397,131 | 1,477 | 5,470 | | | 13,116 | 61,564 | 51,326 | 130,818 | 23,167 | 199,279 | | |
| Stillbirth | 6,331 | 201,339 | 70 | 5,470 | 87 | 3,488 | 223 | 61,563 | 5,951 | 130,818 | | | | |
| Very preterm birth | 1,107 | 76,576 | 82 | 6,440 | | | 940 | 64,670 | 85 | 5,466 | | | | |
| Preterm birth | 18,225 | 267,497 | | | 414 | 7,484 | 2,892 | 48,159 | 406 | 5,403 | | | 14,513 | 206,451 |
| Post-term birth | 27,268 | 391,392 | | | 126 | 7,484 | 4,543 | 48,160 | 424 | 5,360 | 7,049 | 221,186 | 15,126 | 109,202 |
| Low birth weight | 19,180 | 263,971 | 293 | 6,858 | 563 | 7,374 | 2,038 | 69,079 | 16,286 | 180,660 | | | | |
| High birth weight | 6,679 | 260,156 | 123 | 6,858 | 64 | 3,560 | 3,244 | 69,078 | 3,248 | 180,660 | | | | |
| Small for gestational age | 7,448 | 88,857 | 566 | 6,706 | 1,114 | 6,907 | 5,303 | 68,765 | 465 | 6,479 | | | | |
| Large for gestational age | 10,468 | 85,837 | 811 | 6,461 | 626 | 7,395 | 7,865 | 66,203 | 1,166 | 5,778 | | | | |

**Table S3B. Sample sizes contributing to continuous adverse pregnancy and perinatal outcomes with maternal genetic data, across MR-PREG collaboration cohorts and publicly available GWAS.**

| Outcomes | Overall | ALSPAC | BiB | MoBa | UK Biobank | FinnGen | GWAS |
|---|---|---|---|---|---|---|---|
| Birthweight | 289,846 | 7,273 | 8,023 | 74,361 | 200,189 | | |

**Table S4. Description of variables used in ALSPAC observational multivariable regression models**

| Covariate | Measurement | Coding |
|---|---|---|
| Age at menarche | Self-reported in years at three questionnaires:<br>12 week's gestation, when child was 8 years 1 month, and when child was 11 years 2 months.<br>Earliest report used if this was answered at multiple time points. | Continuous in years, with 9 and younger, and 16 and older grouped. |
| Highest educational attainment | Self-reported questionnaire at 32 weeks' gestation. | Categoric: CSE / Vocational / O-level / A-level / Degree. |
| Ethnicity | Self-reported questionnaire at 32 weeks' gestation. | Binary: white / non-white grouped. |
| Age at delivery | Linked NHS delivery records.<br><br>Lower and upper age ranges previously grouped for confidentiality. | Continuous in years. |
| Parity | Self-reported questionnaire at 18 weeks' gestation. | Continuous, with 4 or more grouped. |
| Offspring sex at birth | Linked NHS delivery records. | Binary. |
| Pre-pregnancy BMI | Self-reported questionnaire at 12 weeks' gestation. | Continuous in $kg/m^2$. |

**Table S5. Outcome sample sizes within ALSPAC sample.**

| Outcome | Cases | Controls | Sample size |
|---|---|---|---|
| Any hypertensive disorders of pregnancy | 1470 | 7573 | 9043 |
| Gestational diabetes mellitus | 44 | 9313 | 9357 |
| Gestational hypertension | 1289 | 7573 | 8862 |
| High birthweight | 168 | 8827 | 8995 |
| Large for gestational age | 1064 | 8264 | 9328 |
| Low birthweight | 335 | 8827 | 9162 |
| Perinatal depression | 596 | 7923 | 8519 |
| Post-term birth | 691 | 8326 | 9017 |
| Preeclampsia | 181 | 7573 | 7754 |
| Preterm birth | 424 | 8326 | 8750 |
| Small for gestational age | 663 | 8665 | 9328 |
| Very preterm birth | 91 | 8326 | 8417 |
| Birthweight | NA | NA | 9329 |

Sample sizes available after restricting to complete cases for the outcome of interest. For related outcomes (preterm birth and very preterm; preeclampsia and gestational hypertension), the control definition is the same and so numbers of controls are the same.

**Table S6. ALSPAC participant baseline characteristics by age at menarche[1]**

| Characteristic | Early<br>N = 1,826 | Intermediate<br>N = 6,397 | Late<br>N = 1,218 |
|---|---|---|---|
| Maternal age at delivery | 28.09 (4.92) | 28.49 (4.77) | 28.88 (4.78) |
| Pre-pregnancy BMI | 24.14 (4.47) | 22.74 (3.61) | 22.15 (3.47) |
| Offspring sex | | | |
| Male | 950 (52.0%) | 3,237 (50.6%) | 647 (53.1%) |
| Female | 876 (48.0%) | 3,160 (49.4%) | 571 (46.9%) |
| Parity | | | |
| 0 | 806 (44.1%) | 2,929 (45.8%) | 536 (44.0%) |
| 1 | 624 (34.2%) | 2,295 (35.9%) | 440 (36.1%) |
| 2 | 277 (15.2%) | 841 (13.1%) | 174 (14.3%) |
| 3 | 81 (4.4%) | 256 (4.0%) | 45 (3.7%) |
| 4≤ | 38 (2.1%) | 76 (1.2%) | 23 (1.9%) |
| Ever smoked | | | |
| Yes | 603 (33.0%) | 2,138 (33.4%) | 392 (32.2%) |
| No | 668 (36.6%) | 2,572 (40.2%) | 475 (39.0%) |
| Missing | 555 (30.4%) | 1,687 (26.4%) | 351 (28.8%) |
| Alcohol use before pregnancy | | | |
| Never | 132 (7.2%) | 407 (6.4%) | 113 (9.3%) |
| <1 glass per week | 697 (38.2%) | 2,357 (36.8%) | 443 (36.4%) |
| 1+ glass per week | 785 (43.0%) | 2,833 (44.3%) | 537 (44.1%) |
| 1-2 glasses per day | 171 (9.4%) | 672 (10.5%) | 97 (8.0%) |
| ≥3 glasses per day | 35 (1.9%) | 105 (1.6%) | 21 (1.7%) |
| Missing | 6 (0.3%) | 23 (0.4%) | 7 (0.6%) |
| Education | | | |
| CSE | 338 (18.5%) | 1,006 (15.7%) | 282 (23.2%) |
| Vocational | 145 (7.9%) | 607 (9.5%) | 134 (11.0%) |
| O-level | 701 (38.4%) | 2,259 (35.3%) | 388 (31.9%) |
| A-level | 427 (23.4%) | 1,548 (24.2%) | 275 (22.6%) |
| Degree | 215 (11.8%) | 977 (15.3%) | 139 (11.4%) |
| Ethnicity | | | |
| White | 1,779 (97.4%) | 6,265 (97.9%) | 1,188 (97.5%) |

| | | | |
|---|---|---|---|
| Non-White | 47 (2.6%) | 132 (2.1%) | 30 (2.5%) |

[1]Early age at menarche defined as less than one standard deviation below the mean (11 and younger), intermediate within one standard deviation of the mean (12 to 14 inclusive), and late age at menarche as more than one standard deviation above the mean (15 and older). Mean (SD) for continuous variables, counts (percentages) for categoric variables. Table includes all complete cases (defined as data available on age at menarche, at least one outcome, and all model covariates; n = 9,441).

**Table S7. MR-Egger intercept values testing for evidence of directional pleiotropy for estimated effects of age at menarche on all outcomes.**

| Outcome | MR Egger intercept | Standard error | *P*-value |
|---|---|---|---|
| Hypertensive disorders of pregnancy | -0.00228 | 0.00183 | 0.213 |
| Gestational hypertension | -0.000885 | 0.00207 | 0.669 |
| Preeclampsia | -0.00188 | 0.00219 | 0.392 |
| Gestational diabetes mellitus | -0.00069 | 0.00192 | 0.719 |
| Perinatal depression | -0.00309 | 0.00185 | 0.0963 |
| Low birth weight | -0.0011 | 0.00187 | 0.557 |
| High birth weight | -0.00193 | 0.0029 | 0.506 |
| Very pre-term birth | -0.0149 | 0.00598 | 0.0131 |
| Pre-term birth | -0.00223 | 0.00162 | 0.168 |
| Post-term birth | -0.000316 | 0.00159 | 0.842 |
| Small for gestational age | -0.000372 | 0.00252 | 0.883 |
| Large for gestational age | -0.00252 | 0.00251 | 0.316 |
| Birthweight | -0.000232 | 0.000597 | 0.697 |

**Table S8. Cochran's Q-statistics testing for evidence of between-SNP heterogeneity of effects for MR IVW and multivariable MR (MVMR) estimated effects of age at menarche on all outcomes.**

| Outcome | MR IVW | | MVMR | |
|---|---|---|---|---|
| | Q-statistic | *P*-value | Q-statistic | *P*-value |
| Birthweight | 1075.4 | $1.28x10^{-51}$ | 1060 | $2.14x10^{-55}$ |
| Gestational diabetes mellitus | 803.9 | $9.82x10^{-23}$ | 753 | $2.32x10\text{-}^{21}$ |
| Gestational hypertension | 803.0 | $2.17x10^{-22}$ | 742 | $5.64x10^{-20}$ |
| High birth weight | 587.2 | $2.67x10^{-05}$ | 542 | 0.000152 |
| Hypertensive disorders of pregnancy | 925.7 | $2.68x10^{-35}$ | 832 | $1.93x10^{-29}$ |
| Large for gestational age | 594.4 | $5.35x10^{-07}$ | 539 | $1.38x10^{-05}$ |
| Low birth weight | 677.6 | $1.21x10^{-10}$ | 646 | $6.16x10^{-11}$ |
| Perinatal depression | 550.4 | 0.000526 | 530 | 0.000209 |
| Post-term birth | 615.1 | $8.82x10^{-07}$ | 605 | $3.97x10^{-08}$ |
| Pre-term birth | 781.5 | $1.27x10^{-19}$ | 676 | $1.42x10^{-13}$ |
| Preeclampsia | 838.9 | $5.07x10^{-25}$ | 752 | $2.93x10^{-20}$ |
| Small for gestational age | 459.6 | 0.20 | 428 | 0.252 |
| Very pre-term birth | 448.6 | 0.353 | 424 | 0.315 |

**Table S9. Sample overlap between age at menarche exposure GWAS (n=632,955) and meta-analysed outcome GWAS, by outcome.**
Overlap was due to inclusion of ALSPAC and UK Biobank in the age at menarche GWAS, since these cohorts both contribute to several meta-analysed outcome GWAS.

| Outcome | Outcome GWAS sample size | Maximum overlap | Exposure GWAS in outcome GWAS (%) | Outcome GWAS in exposure GWAS (%) |
|---|---|---|---|---|
| Gestational diabetes mellitus | 714899 | 0 | 0.0 | 0.0 |
| Preeclampsia | 671992 | 0 | 0.0 | 0.0 |
| Any hypertensive disorders of pregnancy | 541768 | 178706 | 33.0 | 28.2 |
| Gestational hypertension | 527932 | 178547 | 33.8 | 28.2 |
| Preterm birth | 418660 | 0 | 0.0 | 0.0 |
| Birthweight | 289846 | 207462 | 71.6 | 32.8 |
| Preterm birth | 285722 | 0 | 0.0 | 0.0 |
| Low birthweight | 283151 | 204097 | 72.1 | 32.2 |
| High birthweight | 266835 | 190889 | 71.5 | 30.2 |
| Perinatal depression | 115797 | 0 | 0.0 | 0.0 |
| Small for gestational age | 96305 | 14216 | 14.8 | 2.2 |
| Large for gestational age | 96305 | 14216 | 14.8 | 2.2 |
| Very preterm birth | 77683 | 12073 | 15.5 | 1.9 |

**Table S10. Sample overlap between pre-pubertal body size exposure GWAS (n=453,169) and meta-analysed outcome GWAS, by outcome.**
Overlap occurred since this pre-pubertal body size GWAS was conducted in UK Biobank only, and the UK Biobank cohort contributes to several meta-analysed outcome GWAS.

| Outcome | Outcome GWAS sample size | Maximum overlap | Exposure GWAS in outcome GWAS (%) | Outcome GWAS in exposure GWAS (%) |
|---|---|---|---|---|
| Gestational diabetes mellitus | 714899 | 0 | 0.0 | 0.0 |
| Preeclampsia | 671992 | 93381 | 13.9 | 20.6 |
| Any hypertensive disorders of pregnancy | 541768 | 93382 | 17.2 | 20.6 |
| Gestational hypertension | 527932 | 93382 | 17.7 | 20.6 |
| Preterm birth | 418660 | 3146 | 0.8 | 0.7 |
| Birthweight | 289846 | 108897 | 37.6 | 24.0 |
| Preterm birth | 285722 | 3160 | 1.1 | 0.7 |
| Low birthweight | 283151 | 107133 | 37.8 | 23.6 |
| High birthweight | 266835 | 100041 | 37.5 | 22.1 |
| Perinatal depression | 115797 | 0 | 0.0 | 0.0 |
| Small for gestational age | 96305 | 3777 | 3.9 | 0.8 |
| Large for gestational age | 96305 | 3777 | 3.9 | 0.8 |
| Very preterm birth | 77683 | 3020 | 3.9 | 0.7 |

**Table S11. Leave-one-study-out Mendelian randomization estimates for birthweight**

Analyses leaving out both UK Biobank (UKB) & ALSPAC aim to address study overlap, since these are the overlapping studies between exposure and outcome GWAS. Both MR IVW (main analysis) and multivariable MR (MVMR) models are presented.

| Analysis | Study left out | Beta (95% CI) | Sample size |
|---|---|---|---|
| MR IVW | UKB left out | 0.013 (-0.007 - 0.033) | 89674 |
| MR IVW | UKB & ALSPAC left out | 0.013 (-0.008 - 0.034) | 82401 |
| MR IVW | MOBA left out | 0.003 (-0.013 - 0.019) | 215485 |
| MR IVW | BIB-WE left out | 0.007 (-0.006 - 0.020) | 286046 |
| MR IVW | BIB-SA left out | 0.010 (-0.003 - 0.023) | 285657 |
| MR IVW | ALSPAC left out | 0.008 (-0.006 - 0.021) | 282590 |
| MR Multivariable, adiposity adjusted | UKB left out | 0.043 (0.021 - 0.066) | 89615 |
| MR Multivariable, adiposity adjusted | UKB & ALSPAC left out | 0.040 (0.016 - 0.064) | 82342 |
| MR Multivariable, adiposity adjusted | MOBA left out | 0.019 (0.000 - 0.038) | 215485 |
| MR Multivariable, adiposity adjusted | BIB-WE left out | 0.028 (0.013 - 0.043) | 285987 |
| MR Multivariable, adiposity adjusted | BIB-SA left out | 0.032 (0.017 - 0.047) | 285598 |
| MR Multivariable, adiposity adjusted | ALSPAC left out | 0.027 (0.011 - 0.042) | 282531 |

**Table S12. Fetal genotype adjusted Mendelian randomization estimates for birthweight**
Effect estimates for a one-year increase in age at menarche on birthweight (SD) adjusted for fetal genotype, compared to unadjusted estimates. Both MR IVW and multivariable MR (MVMR) models are presented.

| Mendelian Randomization model | Approach | Beta (95% CI) |
|---|---|---|
| MR IVW | Unadjusted (main) | 0.007 (-0.009 - 0.022) |
| MR IVW | Adjusted for fetal genotype | -0.008 (-0.026 - 0.010) |
| MR Multivariable, adiposity adjusted | Unadjusted (main) | 0.026 (0.008 - 0.044) |
| MR Multivariable, adiposity adjusted | Adjusted for fetal genotype | 0.006 (-0.014 - 0.026) |

**Table S13. Cochran's Q-statistics testing for evidence of between-SNP heterogeneity of effects for MR IVW and multivariable MR (MVMR) estimated effects of age at menarche on all outcomes, after accounting for fetal genetic effects.**

| Outcome | MR IVW (fetal genotype adjusted) | | MVMR (fetal genotype adjusted) | |
|---|---|---|---|---|
| | Q-statistic | *P*-value | Q-statistic | *P*-value |
| Birthweight | 546 | $1.1x10^{7}$ | 651 | $1.72x10^{-11}$ |
| Gestational diabetes mellitus | 293 | 0.942 | 410 | 0.469 |
| Gestational hypertension | 439 | 0.000429 | 543 | $3.64x10^{-6}$ |
| High birth weight | 382 | 0.164 | 435 | 0.188 |
| Hypertensive disorders of pregnancy | 489 | $3.52x10^{-7}$ | 572 | $4.51x10^{-8}$ |
| Large for gestational age | 383 | 0.0905 | 455 | 0.0532 |
| Perinatal depression | 390 | 0.109 | 437 | 0.157 |
| Post-term birth | 403 | 0.151 | 443 | 0.179 |
| Preterm birth | 493 | $8.6x10^{-6}$ | 511 | 0.000765 |
| Preeclampsia | 529 | $6.88x10^{-6}$ | 581 | $7.06x10^{-7}$ |
| Small for gestational age | 362 | 0.256 | 435 | 0.181 |
| Gestational diabetes mellitus | 293 | 0.942 | 410 | 0.469 |

**Table S14. Genetic variants with known biological roles in pubertal timing, used as instrument for sensitivity analysis.**
SNP = single nucleotide polymorphism. SNP positions based on genome reference build hg37 (as used throughout manuscript).

| **Pathway** | **Gene** | **SNP** | **Chromosome** | **Position** |
|---|---|---|---|---|
| HPG axis disrupted in rare monogenic disorders of puberty | *FEZF1* | rs1899689 | 7 | 121964349 |
| | *GNRH1* | rs6185 | 8 | 25280800 |
| Sex hormone secretion and gametogensis | *HSD17B7* | rs2343506 | 1 | 162892924 |
| | *INHBA* | rs1079866 | 7 | 41470093 |
| | *PCSK2* | rs852061 | 20 | 17109159 |
| Disrupted in rare monogenic disorders of hypogonadism | *SNRPN* | rs8040272 | 15 | 24824016 |

**Table S15. Likelihood ratio tests comparing nested models of early, intermediate, and late categories of age at menarche for each outcome.[1]**

| Outcome | Log likelihood linear model | Log likelihood categoric model | Chi squared test statistic | Degrees of freedom | *P*-value |
|---|---|---|---|---|---|
| Any hypertensive disorders of pregnancy | -3734.54 | -3733.57 | 1.94 | 1 | 0.164 |
| Gestational hypertension | -3434.38 | -3433.78 | 1.21 | 1 | 0.272 |
| Preeclampsia | -787.9 | -787.05 | 1.71 | 1 | 0.192 |
| Preterm birth | -1676.45 | -1676.01 | 0.87 | 1 | 0.352 |
| Very preterm birth | -493.05 | -493.04 | 0.02 | 1 | 0.895 |
| Small for gestational age | -2338.68 | -2338.23 | 0.89 | 1 | 0.347 |
| Low birthweight | -1407.74 | -1407.55 | 0.38 | 1 | 0.540 |
| Gestational diabetes mellitus | -264.64 | -264.17 | 0.94 | 1 | 0.332 |
| Post-term birth | -2422.99 | -2420.95 | 4.09 | 1 | 0.043 |
| Large for gestational age | -3180.07 | -3178.2 | 3.75 | 1 | 0.053 |
| High birthweight | -770.41 | -770.38 | 0.05 | 1 | 0.822 |
| Perinatal depression | -2113.69 | -2111.83 | 3.73 | 1 | 0.053 |
| Birthweight | -12524.42 | -12523.65 | 1.55 | 1 | 0.214 |

[1]Early age at menarche defined as less than one standard deviation below the mean (11 and younger), intermediate within one standard deviation of the mean (12 to 14 inclusive), and late age at menarche as more than one standard deviation above the mean (15 and older). All models were fully adjusted for all covariates (age at delivery, parity, offspring sex, educational attainment, ethnicity, adiposity). Linear model degrees of freedom = 11, Categoric model degrees of freedom = 12.
